# Genome-wide identification of *XTH* genes in *Liriodendron chinense* and functional characterization of *LcXTH21*

**DOI:** 10.3389/fpls.2022.1014339

**Published:** 2022-10-27

**Authors:** Junpeng Wu, Yaxian Zong, Zhonghua Tu, Lichun Yang, Wei li, Zhengkun Cui, Ziyuan Hao, Huogen Li

**Affiliations:** Key Laboratory of Forest Genetics & Biotechnology of Ministry of Education, Co-Innovation Center for Sus-tainable Forestry in Southern China, College of Forestry, Nanjing Forestry University, Nanjing, China

**Keywords:** *Liriodendron chinense*, XTH family, drought stress response, genome identification, root development

## Abstract

*Liriodendron chinense* is a relic tree species of the family Magnoliaceae with multiple uses in timber production, landscape decoration, and afforestation. *L. chinense* often experiences drought stress in arid areas. However, the molecular basis underlying the drought response of *L. chinense* remains unclear. Many studies have reported that the xyloglucan endotransglucosylase/hydrolase (*XTH*) family plays an important role in drought stress resistance. Hereby, to explore the drought resistance mechanism of *L. chinense*, we identify *XTH* genes on a genome-wide scale in *L. chinense*. A total of 27 *XTH* genes were identified in *L. chinense*, and these genes were classified into three subfamilies. Drought treatment and RT-qPCR analysis revealed that six *LcXTH* genes significantly responded to drought stress, especially *LcXTH21*. Hence, we cloned the *LcXTH21* gene and overexpressed it in tobacco *via* gene transfer to analyze its function. The roots of transgenic plants were more developed than those of wild-type plants under different polyethylene glycol (PEG) concentration, and further RT-qPCR analysis showed that *LcXTH21* highly expressed in root compared to aboveground organs, indicating that *LcXTH21* may play a role in drought resistance through promoting root development. The results of this study provide new insights into the roles of *LcXTH* genes in the drought stress response. Our findings will also aid future studies of the molecular mechanisms by which *LcXTH* genes contribute to the drought response.

## Introduction

Plants are continuously exposed to various types of abiotic stress because they are sessile, and drought stress has a negative effect on the growth, yield, and cultivation of plants. Given that water scarcity reduces plant performance, improving the drought resistance of plants is a major goal of breeding efforts (Boyer, 1982; Bray et al., 2000; Cushman and Bohnert, 2000; Mittler, 2006). Plant improvement of drought resistance *via* molecular breeding approaches is a clear trend that further promotes the development of modern agriculture. With the various genome resources available, mining and utilizing genes that provide high resistance to drought stress will promote the development of plant molecular breeding (Parmar et al., 2017; Wai et al., 2020).

In recent years, identification and functional analysis of various abiotic stress-responsive genes and transcription factors and their applications in breeding stress-tolerant plants were favored (Roy et al., 2011; Sharma et al., 2017). For example, overexpression of *HhGRAS14* in *Arabidopsis thaliana* significantly improved the drought tolerance of transgenic plants (Ni et al., 2022). And orphan gene *PpARDT* was found to be involved in drought tolerance potentially by enhancing ABA response in *Physcomitrium patens* (Dong et al., 2022). *MdFLP* enhanced drought tolerance by regulating the expression of *MdNAC019* in self-rooted apple stocks (Wang et al., 2022). The bZIP transcription factor ABP9 in maize is involved in the regulation of drought resistance, and overexpression of *OsNAC10* in rice increased grain yield under drought stress (Jeong et al., 2010; Wang et al., 2017). The overexpression of a rice *OsSalT* in tobacco showed increased root growth and resulted in improved drought tolerance (Kaur et al., 2022). In *Manihot esculenta*, *MeRSZ21b* was found to be involved in drought tolerance, and plants overexpressed *MeRSZ21b* gene had longer roots than WT (Chen et al., 2022).

Generally, there are gene families related to abiotic stress in plant genomes. The xyloglucan endotransglucosylase/hydrolase (*XTH*) family is a typical example (Eklöf and Brumer, 2010). *XTH* family belongs to the glycoside hydrolase 16 family (GH16), and *XTH* genes can be divided into four groups: I/II III-A, III-B, and the early diverging group (Campbell and Braam, 1999b). Increasing evidence has revealed that genes in the *XTH* family play an important role in drought stress. For example, plants overexpressing *GmXTH23* had stronger drought tolerance and greater root lengths than wild-type (WT) plants (Long, 2020). Following overexpression of the hot pepper gene *CaXTH3* in tomato, half of the stomata of transgenic plants were open under drought stress, and most of the stomata of WT plants were closed, a condition that was suggestive of transgenic plants having a higher drought tolerance than WT plants (Choi et al., 2011). *XTH* genes have also been shown to be involved in plant growth. For example, GUS staining of *A. thaliana* has shown that *AtXTH* genes might be expressed throughout all growth stages (Becnel et al., 2006). The expression levels of *DcXTH2* and *DcXTH3* increased dramatically during flowering, confirming that *XTH* genes play a role in petal growth (Harada et al., 2011). In addition, some plants use xyloglucan as a storage polysaccharide for embryonic development (Reid, 1985; Buckeridge et al., 2000). Due to their important roles in drought stress and growth, *XTH* family members have been identified in various plants, including *A. thaliana* (33 genes), *Oryza sativa* (29 genes), *Hordeum vulgare* (24 genes), *Populus* spp. (41 genes), *Ananas comosus* (24 genes), and *Schima superba* (34 genes) (Yokoyama and Nishitani, 2001; Yokoyama et al., 2004; Geisler-Lee et al., 2006; Fu et al., 2019; Li et al., 2019; Yang et al., 2022).

*L. chinense* is a relict species in the Magnoliaceae family that has been widely used in timber production, landscape decoration, and afforestation. Drought stress has become a major barrier restricting the cultivation of *L. chinense* (He and Hao, 1999). As mentioned above, *XTH* family has been widely reported to be involved in drought response. However, the precise role and molecular mechanisms of the *XTH* family under drought stress remains unclear in *L. chinense*. Hereby, in order to have a better insight into the roles of *XTH* genes in *L. chinense*, we identified *LcXTH* genes on a genome-wide scale, uncovered *LcXTH* genes in relation to drought resistance and characterized their function. Overall, our study provides new insights into the possible roles of *XTH* genes in *L. chinense* and will aid future studies of drought resistance mechanisms in woody plants.

## Materials and methods

### Plant materials and growth conditions

*L. chinense* material used in this study was obtained from Xiashu Forest Station at Nanjing Forestry University, Jurong, Jiangsu, China. In April 2021, leaves, roots and leaf buds were collected from a 30-year-old *L. chinense* tree originating form Songyang, Zhejiang Province, and immediately frozen in liquid nitrogen, and then stored at –80°C until further use. *L. chinense* seeds were soaked in water for 2 days, transplanted to soil, and cultivated in 40 cm × 30 cm × 4 cm trays for 2 months with a 16-h/8-h light/dark photoperiod. All *L. chinense* seedlings were watered twice a week. To investigate the response of *L. chinense* to drought stress, seedlings were watered with 10% PEG (100 g/L) solution. Samples of *L. chinense* were taken at 0, 3, 6, 12, 24, and 48 h and immediately frozen in liquid nitrogen for RT-qPCR analysis. All seedlings were grown under the same conditions, with the exception of plants in the drought stress treatment.

Tobacco (*Nicotiana benthamiana*) was used for transgenic assays. Before sowing, seeds were sterilized with 75% (v/v) alcohol for 30 s, NaClO (v/v) for 15 min, and double-distilled water four times. Seeds were then sown on 1/2 MS medium (Murashige and Skoog, 2006) and vernalized at 4°C in the dark for 2 days. These seeds were placed in an incubator (SANYO, Japan) under a photoperiod 16-h/8-h light/dark photoperiod at 23°C for 10 days; they were then transplanted into MS medium and cultivated for 20 days. WT tobacco was used as a control, and all seedlings were grown in the same environment. The medium of the 30-day-old transgenic and WT tobacco seedlings was removed, and seedlings were planted in trays. Transgenic and WT tobacco plants were treated with 10% PEG (100 g/L) solution for 5 days, and root length was measured.

### Identification and characterization of *XTH* family members in *L. chinense*

The two XTH domains (PF00722 and PF06955) were used as queries to search the *L. chinense* genome with HMMER (v. 3.0). The default settings and cutoff values were set to 0.001 (Potter et al., 2018; El-Gebali et al., 2019). Potential sequences were filtered using the Conserved Domain Search Service website (https://www.ncbi.nlm.nih.gov/Structure/bwrpsb/bwrpsb.cgi) (Marchler-Bauer and Bryant, 2004), and the candidate genes were identified using the SMART database (https://smart.embl.de/) (Letunic et al., 2021). Redundant genes were removed manually. The molecular weight (MW), isoelectric point (pI), and protein length were analyzed using the ExPASy website (https://web.expasy.org/protparam/) (Wilkins et al., 1999). Single peptides and the subcellular localization of *LcXTH* genes were predicted by SignalP (https://dtu.biolib.com/SignalP-6) and Plant-mPLoc (v. 2.0) (http://www.csbio.sjtu.edu.cn/bioinf/plant-multi/), respectively (Chou and Shen, 2010; Teufel et al., 2022). The Blast program was used to identify homologous genes (Chen et al., 2020).

### Chromosomal localization, synteny analysis, and tandem repeat analysis

Information on the chromosomal location of *LcXTH* genes was obtained from the genome GFF file. Ka and Ks values, protein similarity matrices, and collinear gene pairs were analyzed using TBtools (Chen et al., 2020).

### Phylogenetic analysis of *LcXTH* genes

*A. thaliana* proteins were downloaded from the TAIR website (https://www.A.thaliana.org/). *H. vulgare* proteins, *O. sativa* proteins were download from NCBI website (https://www.ncbi.nlm.nih.gov/). The sequences were aligned with ClustalW software (v. 2.1). A phylogenetic tree was constructed by MEGA 7.0 software using the neighbor-joining method with the following parameters: Poisson model, pairwise deletion, and 1000 bootstrap replicates (Kumar et al., 2016). The evolview website (https://www.evolgenius.info/evolview-v2/) was used to modify the phylogenetic tree (Subramanian et al., 2019).

### Expression pattern analysis and GO annotation

The expression levels of *LcXTH* genes were evaluated using fragments per kilobase of transcript per million mapped reads (FPKM) values based on transcriptome data (https://www.ncbi.nlm.nih.gov/, PRJNA559687) from different tissues of *L. chinense*, and heat maps were constructed using TBtools (Chen et al., 2020). Gene Ontology (GO) analysis was performed using the clusterProfiler 4.0 (Wu et al., 2021).

### Analysis of motifs and gene structure

The online software MEME (https://meme-suite.org/meme/doc/meme.html) was used to analyze the conserved motifs of XTH proteins with a maximum of 10 motifs (Bailey et al., 2015). The Gene Structure Display Server (http://gsds.gao-lab.org/) was used to analyze gene structure (Hu et al., 2014). The promoter sequence (2000 bp upstream of the start codon) of each *LcXTH* gene was extracted and then analyzed using PlantCARE online software (http://bioinformatics.psb.ugent.be/webtools/plantcare/html/); the results were visualized using TBtools. The protein sequences were submitted to ESPript Web server (http://espript.ibcp.fr/ESPript/ESPript/) for secondary structure prediction.

### Extraction of RNA and RT-qPCR analysis

A SteadyPure Plant RNA Extraction Kit (AG21019, Accurate Biotechnology, Hunan, Co., Ltd.) was used for RNA extraction following the user manual. The quality of RNA was assessed using a NanoDrop 2000 spectrophotometer. A260/A280 values ranged from 1.8 to 2.0, and values of A260/A230 ranged from 1.9 to 2.1; a total of 500 ng of RNA was used to synthesize complementary DNA (cDNA).

RT-qPCR was used to analyze the expression profiles of *LcXTH* genes, and *Actin97* was used as the reference housekeeping gene (Tu et al., 2019). The thermal cycling conditions for RT-qPCR were based on instructions provided in the SYBR Green Premix Pro Taq HS qPCR Kit (AG11701, Accurate Biotechnology, Hunan, Co., Ltd.). We used the 2^−ΔΔCT^ method to calculate relative levels of expression (Livak and Schmittgen, 2001). Three biological replicates and technical replicates were conducted to ensure the accuracy of the results. And primers used in this study are listed in **Table S1**.

### Full-length cDNA cloning of *LcXTH21* and plant transformation

The coding sequence of *LcXTH21* was obtained from the genome of *L. chinense*, and primers were designed using Oligo software (v. 7). The full-length cDNA of *LcXTH21* was amplified, and an 876-bp open reading frame sequence was obtained (**File S1**). The cDNA of *LcXTH21* was then cloned into the modified pBI-121 vector, which was digested with *Xba*I and *Bam*HI QuickCut enzymes (Takara Biomedical Technology, Dalian, China). The transgene construct was introduced into *Agrobacterium tumefaciens* strain GV105, which was then transformed into tobacco using a leaf-disc infection method. We cut off the edge of wild-type tobacco leaves that had been cultured for about 30 days, put them in solid MS medium at 25°C for 2 days in the dark. Then we activated the transformed *A. tumefaciens* with liquid MS medium for 30 min to prepare an infection solution, then immersed the leaves in the infection solution for 10 min. After soaking, the leaves were cultivated continuously in the dark at 25°C for two days, then placed them at 25°C for 16 h in the light and 8 h in the dark. When callus grown on the edge of the leaves, we isolated callus and cultured on MS medium containing kanamycin. After 20 days of culture, PCR was used to determine whether the plants were positive, and positive plants were cultured until they reached maturity. The details can be seen in **Figure S1**.

## Results

### Identification and characteristics of *LcXTH* genes

HMM searches were used to identify *XTH* genes. We originally obtained 29 putative *XTH* genes. These 29 candidate genes were then submitted to the SMART database, and incomplete sequences were removed manually. Finally, 27 *XTH* genes were obtained, which were named *LcXTH1*–*LcXTH27*.

The characteristics and subcellular localization of LcXTH proteins were also predicted. The length of LcXTH proteins ranged from 243 to 337 amino acids, LcXTH26 and LcXTH27 were the largest proteins with 337 amino acids, and LcXTH17 was the smallest (243 aa). The theoretical pI values for LcXTH proteins ranged from 4.85 to 9.65, and the MW of these proteins ranged from 27.61 to 38.47 kDa. All LcXTH members were predicted to be localized to the cell wall, and 15 LcXTH members were predicted to be localized to the cytoplasm. Details are provided in **Table S2**.

### Chromosome location and homology analysis

The chromosomal location of genes is determined by prior evolutionary events. We thus investigated the chromosomal locations of *LcXTH* genes. *LcXTH* genes were randomly distributed on nine chromosomes (**Figure S2**). However, because the genome assembly was incomplete, the specific chromosomal locations could not be determined for two genes: *LcXTH26* and *LcXTH27*. Most *LcXTH* genes were clustered on chromosomes 13, 14, and 17; chromosome 17 had 13 genes; and chromosomes 1, 3, 5, 9, and 16 had only one gene.

Synteny within the *LcXTH* family was analyzed to clarify the evolutionary relationships among *LcXTH* genes, and three homologous pairs (*LcXTH08*-*LcXTH12*, *LcXTH09*-*LcXTH14*, and *LcXTH02*-*LcXTH05*) were identified (**Figure S2**). The identity of *LcXTH08*-*LcXTH12*, *LcXTH09*-*LcXTH14*, and *LcXTH02*-*LcXTH05* was 81.30%, 67.25%, and 82.8%, respectively. The substitution rates (Ka/Ks) of these three gene pairs were calculated to assess whether *LcXTH* genes have been subjected to selection (**Table S3**). The substitution rates ranged from 0.099 to 0.114, which indicated that they have experienced purifying selection. These homologous pairs (*LcXTH08*-*LcXTH12*, *LcXTH09*-*LcXTH14*, and *LcXTH02*-*LcXTH05*) diverged approximately 66.42, 188.19, and 67.38 million years ago, respectively. We also evaluated the density of these genes across the entire genome. The density of these genes was high in regions with related homologous genes.

Tandem duplication is an important mechanism underlying the expansion of gene families, and tandemly duplicated genes often occur in clusters (Kozak et al., 2009). Hence, we calculated the protein similarity matrix to investigate the identity of *LcXTH* genes in three gene clusters (**Figure S3** and **Table S4**). The identity of the *LcXTH* genes on chromosome 13 was 60.54%, and the identity of the three *LcXTH* genes on chromosome 14 ranged between 58.46% and 73.29%. Chromosome 17 contained 13 *LcXTH* genes, and the identity ranged from 47.16% to 96.59%. We speculate that the high identity of these closely arranged genes indicates that they are products of tandem duplication; generally, tandem duplication might be the major force driving the expansion of *LcXTH* genes.

### Phylogenetic analysis of XTH proteins

**Figure 1:**
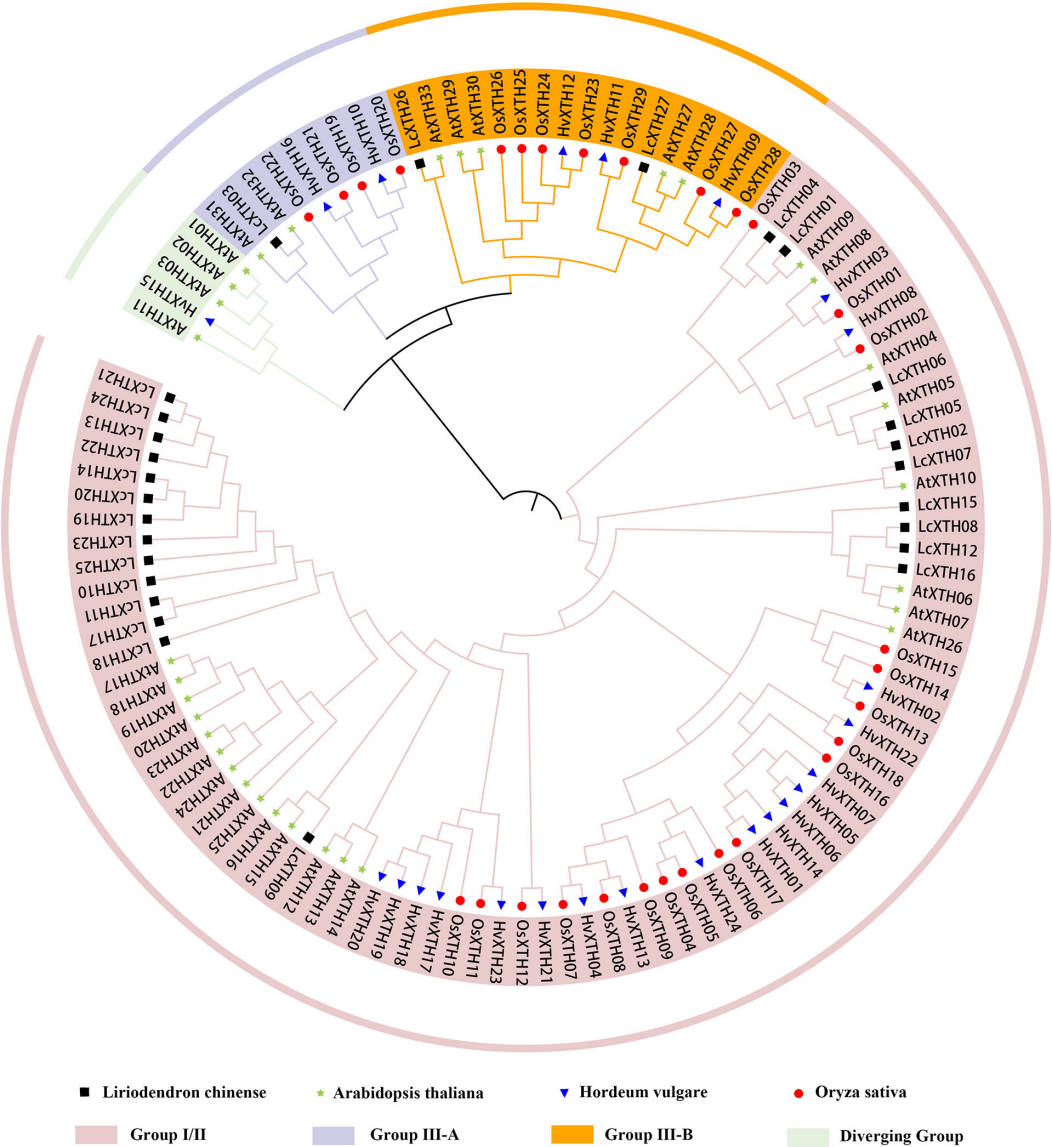
Classification of XTH proteins. The phylogenetic tree was constructed using 27 *Liriodendron chinense* XTH proteins, 24 *Hordeum vulgare* XTH proteins, 29 *Oryza sativa* XTH proteins and 33 *Arabidopsis thaliana* XTH proteins. Branches ending with Black square, blue triangle, red circle, green star indicate *L. chinense*, *H. vulgare*, *O. sativa* and *A. thaliana*, respectively. Proteins with a green, purple, orange and pink background indicate the early diverging group, group III-A, group III-B, and group I/II, respectively.

To further investigate the evolutionary relationships among LcXTH family members, we constructed a phylogenetic tree using 113 XTH proteins. (**Figure 1**). Phylogenetic analysis revealed that all XTH proteins were classified into four groups (group I/II, group III-A, group III-B, and the early diverging group). In *L. chinense*, most *LcXTH* genes (24) were categorized into group I/II. Only one gene (*LcXTH03*) was classified in group III-A. The remaining genes (*LcXTH26* and *LcXTH27*) were grouped into III-B. No genes were classified into the early diverging group in *L. chinense*, which might stem from the incomplete genome annotation or gene loss.

### Structure and motif patterns of *LcXTH* genes

**Figure 2:**
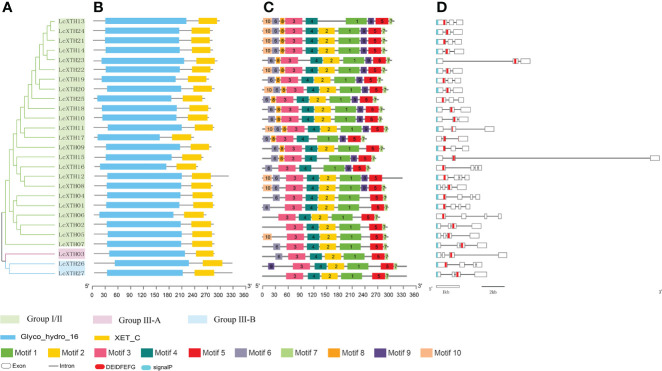
Gene structure and conserved domains of LcXTH proteins. **(A)** The cluster of LcXTH proteins. The green, pink blue background indicate group I/II group III-A and group III-B, respectively. **(B)** Conserved domains were predicted using the SMART database. The blue blocks indicate Glyco_hydro_16 domains, and the yellow blocks indicate XET_C domains **(C)** Conserved motifs in 27 LcXTH proteins were predicted using the MEME tool, and different color blocks correspond to different motifs. **(D)** The exon–intron distribution was visualized using the GSDS 2.0 server, and white boxes, black lines, red blocks, and blue blocks indicate exons, introns, the conserved domain ExDxE, and signal peptides, respectively.

To investigate the structure of LcXTH proteins, we submitted the protein sequences to the SMART database to identify conserved domains. We analyis the pylogenetic analysis of LcXTH proteins and two domains (Glyco_hydro_16 and XET_C) were identified in all LcXTH proteins (**Figure 2A** and **Figure 2B**). XET_C is a unique domain among GH16 family members, and the proteins in this family had a common structure known as the *β*-jellyroll fold (Atkinson et al., 2009; Eklöf and Brumer, 2010; Behar et al., 2018). We also performed conserved motif analysis on LcXTH proteins (**Figure 2C**) and found that the motif composition of LcXTH family members was similar. As shown in the schematic, the Glyco_hydro_16 domain included motifs 10, 6, 8, 3, 4, 2, and 1, and the XET_C domain included motifs 9, 5, and 7. Motifs 1, 3, 4 (ExDxE), and 5 were conserved in all LcXTH proteins.

Previous studies have shown that the exon distribution of *AtXTH* genes is conserved within each subfamily in *A. thaliana* (Yokoyama and Nishitani, 2001; Yokoyama and Nishitani, 2008). We used the online tool GSDS 2.0 to analyze the exon–intron organization of the 27 *LcXTH* genes (**Figure 2D**). There were three or four exons in *LcXTH* genes, and the ExDxE domain was randomly distributed in these genes, with exception of the fourth exon. The signal peptides of LcXTH proteins were predicted. A total of 23 LcXTH proteins had signal peptides, and they were all located on the first exon. These short amino acid sequences might be responsible for transmembrane transport and have secretory functions.

### Structure-based sequence alignment

**Figure 3:**
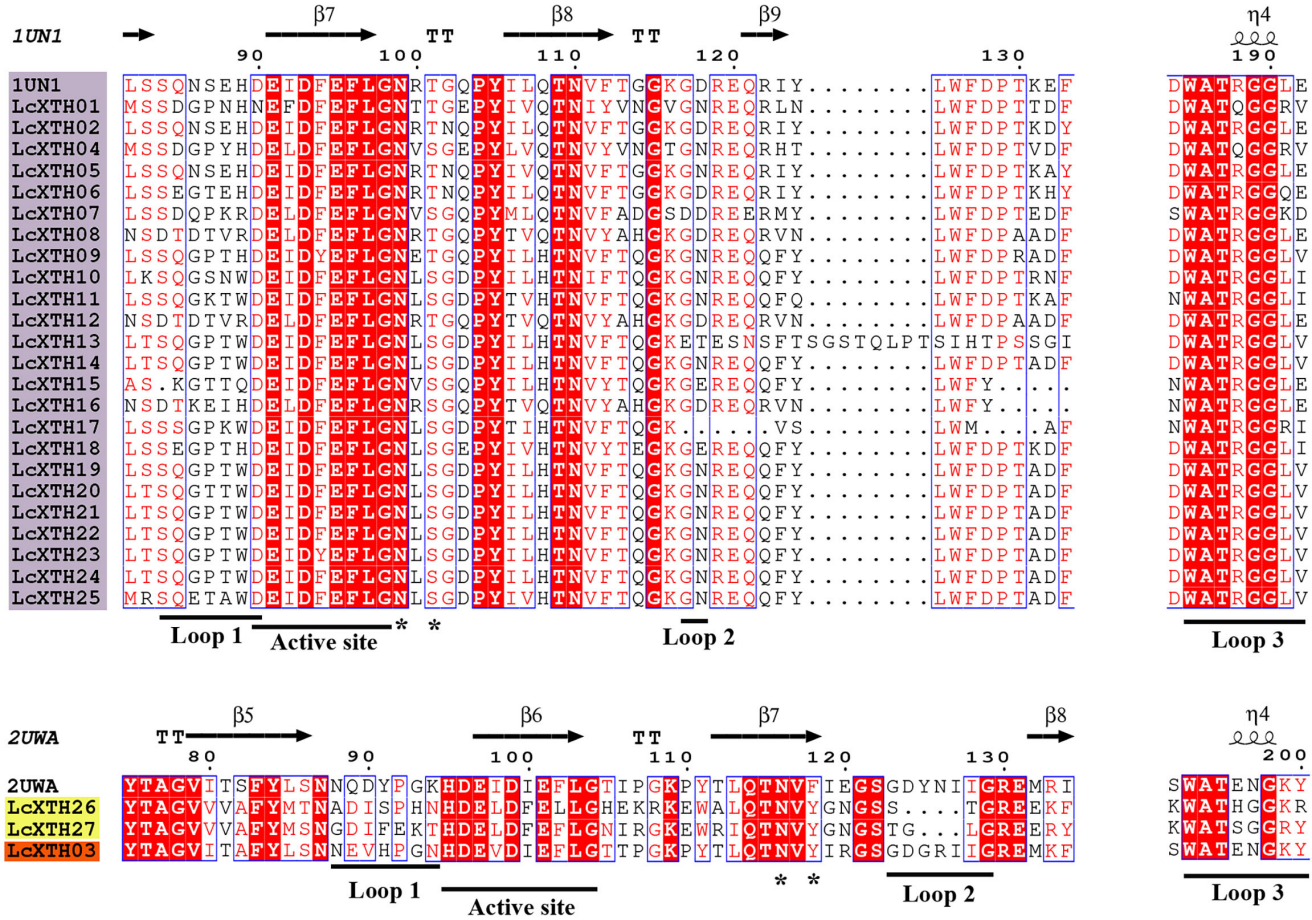
Structure-based sequence alignment of LcXTH proteins. Proteins with a purple background, proteins with a yellow background, and proteins with an orange background indicate Group I/II Group III-B, and Group III-A members, respectively. Blue frames, white letters in red boxes, and red letters in white boxes indicate conserved residues, strict identity, and similarity, respectively. The secondary structures of *β* sheets (arrows), *α*-helices (spiral), N-glycosylation site (*), and loops 1, 2, and 3 (lines) are indicated.

To further characterize LcXTH proteins, two fully resolved structures of PttXET16-34 (PDB ID: 1UN1) and TmNXG1 (PDB ID: 2UWA) were used to characterize the secondary structures of XTH proteins with ESPript software. The schematic of the secondary structures shows that all LcXTH proteins contained the conserved ExDxE domain (**Figure 3**). The first glutamic acid residue (E) acts as a catalyzed nucleophile, which typically initiates enzymatic reactions, and the second E residue acts as a base that activates the entering substrate (Fu et al., 2019). The N-glycosylation domain (NXT/S/Y) is thought to be critical for protein stability and is indicated in the figure. This N-glycosylation site was conserved in all group I/II proteins, but it was missing in nearly all group III-A XTH proteins (Eklöf and Brumer, 2010). However, the N-glycosylation site was observed in all LcXTH members, including LcXTH03 (a member of group III-A). The N-glycosylation sites of LcXTH proteins in group III were shifted by approximately 20 amino acids from the ExDxE domain to the C-terminus.

The architecture of proteins was conserved within specific groups; for example, other conserved domains adjacent to the ExDxE domain were identified in LcXTH proteins, which were referred to as loop 1, loop 2, and loop 3. Previous studies have demonstrated that the extension of loop 2 plays a key role in determining the activity of XTH proteins (Baumann et al., 2007). In this study, Loop 2 was significantly shorter in groups I/II and III-B than in group III-A, suggesting that the difference in the length of loop 2 among subfamilies of *L. chinense* might partly account for the differences in the classification of these proteins and their functions. The sequence DWATRGG of loop 3 was present in most group I/II proteins; however, this sequence was replaced by SWATEN in group III-A members.

### Tissue expression patterns and GO analysis of *LcXTH* genes

**Figure 4:**
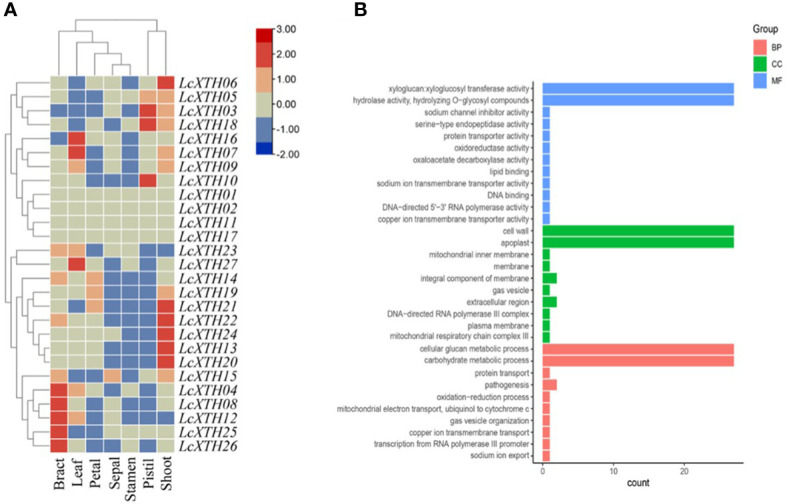
Expression patterns and GO annotations of *LcXTH* genes. **(A)** Heat map of the expression levels of *LcXTH* genes across different tissues in *L. chinense* based on FPKM values from public transcriptomic data. **(B)** GO classification of *LcXTHs*. Details of the GO annotations are provided in **Table S5**.

We analyzed the expression patterns of *LcXTH* genes across several tissues (including bracts, sepals, petals, stamens, pistils, leaves, and shoots) (**Figure 4A**). *LcXTH* genes showed tissue-specific expression patterns. *LcXTH04*, *LcXTH12*, *LcXTH08*, *LcXTH25*, and *LcXTH26* were highly expressed in bracts, and the expression levels of these genes in other tissues were low. *LcXTH07*, *LcXTH16*, and *LcXTH27* were significantly expressed in leaves. The expression levels of *LcXTH03*, *LcXTH18*, and *LcXTH10* were high in pistils, suggesting that they are involved in pistil development. The expression levels of almost all *LcXTH* genes were low in sepals, petals, and stamens, suggesting that *LcXTH* genes were not expressed during flowering. The expression patterns of the three tandem arrays on chromosomes 13, 14, and 17 were not consistent. *LcXTH13*, *LcXTH19*, *LcXTH20*, *LcXTH21*, *LcXTH22*, and *LcXTH24* were significantly expressed in shoots and had similar expression patterns, indicating that their functions might be redundant. However, the expression patterns of the other genes on chromosome 17 differed, suggesting that they might have acquired new functions. In addition, *LcXTH01*, *LcXTH02*, *LcXTH11*, and *LcXTH17* were not expressed in any of the tissues examined in this study; this indicates that they are not expressed or have specific expression patterns that could not be detected in this study.

GO analysis was performed to clarify the functions of *LcXTH* genes. All *LcXTH* genes encoded proteins with xyloglucan xyloglucosyl transferase and hydrolase activity, and they were all localized to the cell wall and the apoplast, which was consistent with the subcellular localization prediction (**Figure 4B**). All *LcXTH* genes were predicted to be involved in cellular glucan metabolic process and carbohydrate metabolic process.

### *Cis*-element prediction and RT-qPCR analysis

**Figure 5:**
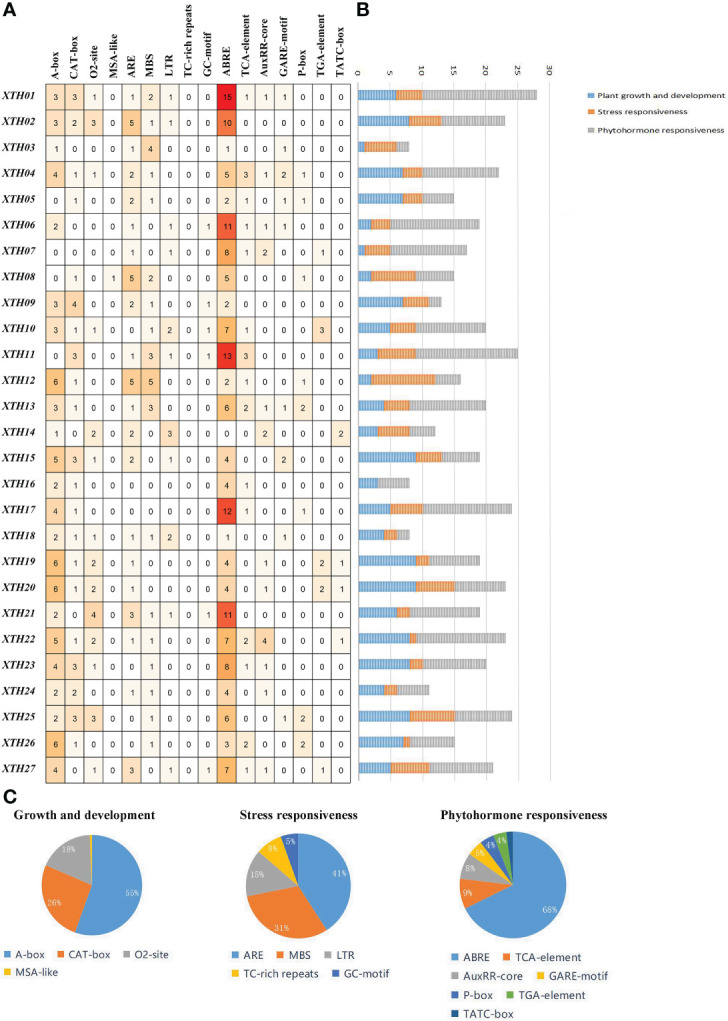
Analysis of *cis*-elements in *LcXTH* promoters. **(A)** Number of distinct *cis*-elements in each *LcXTH*. **(B)** Sum of *cis*-elements for each class of LcXTH proteins. **(C)** Proportion of each item in different categories.

To determine the expression patterns and regulatory characteristics of *LcXTH* genes, the 2000-bp upstream sequence of the translation initiation site (ATG) was extracted, and *cis*-elements were predicted (**Figure 5A**). A large number of *cis*-elements were involved in plant growth and development, stress responses, and phytohormone responses (**Figures 5B**, **C**). In the first category, the main *cis*-elements were motif CAT-box (33.64%), motif CCAAT-box (27.1%), motif O2-site (23.36%), and GCN4-motif (11.21%). *Cis*-elements involved in stress responses mainly included the ARE motif (40.78%), MBS motif (30.1%), LTR motif (14.56%), TC-rich repeats (7.77%), and GC-motif (5.83%). In the third category, the main *cis*-elements were abscisic acid-responsive element (ABRE, 58.48%), methyl jasmonate-responsiveness element (CGTCA, 14.8%), and auxin-responsive element (TGA-element, 7.97%). The most abundant phytohormone response element is associated with abscisic acid (ABA)-responsiveness, indicating that *LcXTH* genes might be regulated by ABA.

**Figure 6:**
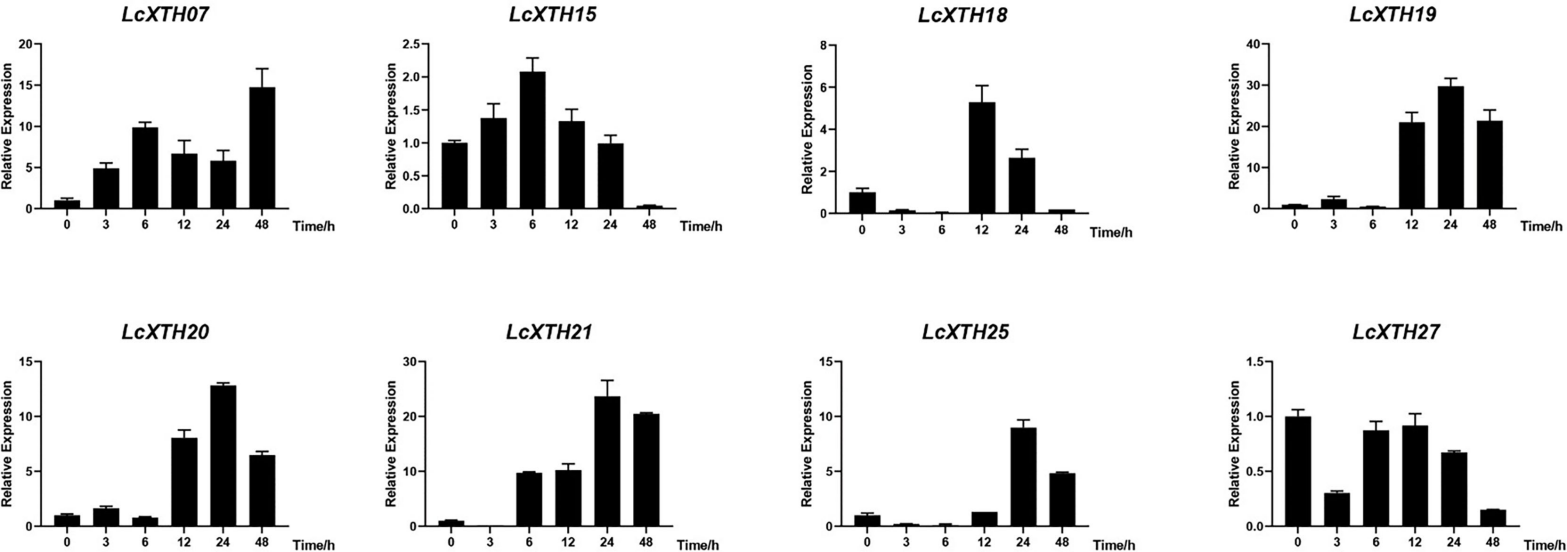
Expression profiles of *LcXTH* genes in different drought stress periods. Three independent experiments were performed using *Actin97* as an internal reference gene. Error bars on the graph indicate the mean standard deviation for each triplicate treatment.

To clarify the roles of *LcXTH* family members in drought resistance, we used BLASTp to identify stress-related *LcXTH* genes based on previous studies, and genes containing plant defense and stress elements (TC-rich motifs) were screened out (Xu et al., 2020). A total of eight *LcXTH* genes (*LcXTH07*, *15*, *18*, *19*, *20*, *21, 25*, and *27*) were identified, and their expression patterns under drought stress were clarified. The RT-qPCR results revealed that there were significant differences in the expression of these genes at different times under drought stress (**Figure 6**). *LcXTH07* expression was up-regulated at 0 and 6 h, down-regulated at 6 and 24 h, and highest at 48 h. Some genes exhibited similar expression patterns. For example, the expression patterns of *LcXTH18, 19*, *20*, and *25* did not change significantly during 0 and 6 h, but significantly increased at 12 h. *LcXTH21* exhibited the most rapid and strongest response to drought stress, and it was the most highly expressed gene. Some genes exhibited opposite expression patterns; the expression levels of *LcXTH15* and *LcXTH27* changed slightly, indicating that they might not play important roles in the response to drought stress.

### Overexpression of *LcXTH21* in tobacco promoted root development under drought stress

**Figure 7:**
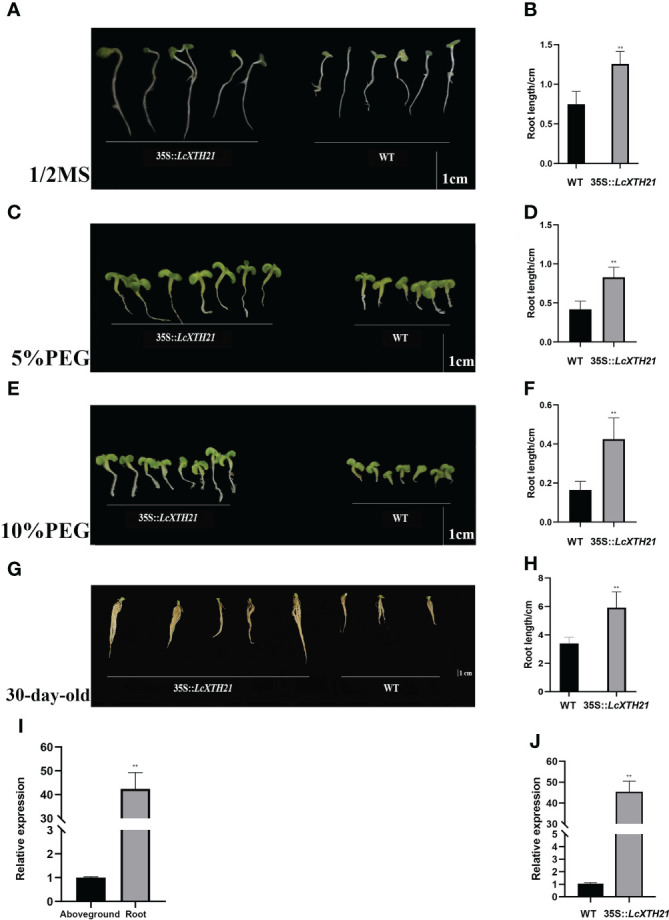
Phenotypic changes caused by *LcXTH21* overexpression in tobacco, and histograms of root length in 10-day-old and 30-day-old transgenic tobacco and WT seedlings under different PEG concentration. **(A)** Comparison of the root length between 10-day-old transgenic tobacco and WT seedlings on 1/2 MS medium. **(B)** Statistical histograms of root length in 10-day-old transgenic tobacco and WT seedlings on 1/2 MS medium. **(C)** Comparison of the root length between 10-day-old transgenic tobacco and WT seedlings under 5% PEG. **(D)** Statistical histogram of root length in 10-day-old transgenic tobacco and WT seedlings under 5% PEG. **(E)** Comparison of the root length between 10-day-old transgenic tobacco and WT seedlings under 10% PEG. **(F)** Statistical histogram of seedling height in 10-day-old transgenic tobacco and WT seedlings under 10% PEG. **(G)** Comparison of the root length between 30-day-old transgenic tobacco and WT seedlings under 10% PEG. **(H)** Statistical histogram of seedling height in 30-day-old transgenic tobacco and WT seedlings under 10% PEG. **(I)** Relative expression of *LcXTH21* between root and aboveground. Bars = 1 cm. **(J)** Relative expression of *NCED* gene between WT and transgenic tobacco. Statistical analyses were performed using *t*-tests (* P < 0.05, ** P < 0.01).

In light of the strong response of *LcXTH21* under drought stress, we cloned *LcXTH21* and overexpressed it in tobacco to analyze its function; the characteristics of transgenic and WT tobacco plants were noted. Then, we cultivated transgenic plants and WT on 1/2 MS medium containing different PEG concentration. With the increase in PEG concentration, the root length decreased to varying degrees. Ten-day-old transgenic plants on 1/2 MS medium had an average root length of 1.26 cm, which was 68% longer than that of WT plants (**Figures 7A, B**). Under 5% PEG, the average root length in transgenic plants decreased by 52%, however, the WT root length decreased by 79% (**Figures 7C, D**). When the PEG concentration was increased to 10%, the average root length of transgenic tobacco and WT decreased by 95% and 154%, respectively (**Figures 7E, F**). Next, we treated thirty-day-old seedlings with 10% PEG for 5 days, and the root length were compared. The average root length was 2.11-fold that of WT plants (**Figures 7G, H**). To investigate the expression level between root and aboveground, we took samples of root and aboveground for RT-qPCR analysis. The results shown that the expression level of root was 42.06-fold than that of aboveground tissues/organs (**Figure 7I**). The plant hormone ABA plays a key role in regulating the resistance of plants to drought stress, whereas the NCED enzyme is a key rate-limiting ABA biosynthetic enzyme (Leung and Giraudat, 1998; Zhang et al., 2009; Zhu et al., 2017). Hence, to further study the relationship of the *LcXTH21* genes and ABA signaling, we performed RT-qPCR analysis to detect the expression of *NCED* gene in thirty-day-old WT and transgenic tobacco (**Figure 7J**). The results showed that the relative expression level of *NCED* in transgenic plants was about 45-fold higher than that in WT controls, thereby suggesting that *LcXTH21* might contribute to drought resistance by promoting ABA biosynthesis.

## Discussion

### The evolution and structure of *LcXTH* family members

The evolution of the *XTH* family has received wide research interest. *XTH* genes were first detected in Zygnematophyceae and non-charophycean taxa; bacterial licheninases have long been considered non-plant ancestors of the *XTH* family because of their sequence similarities (Barbeyron et al., 1998; Michel et al., 2001; Del Bem and Vincentz, 2010; Behar et al., 2018). The expansion of *XTH* genes has also received much research interest. The relaxation of substrate specificity, including the broader specificity of group I/IImembers, might have contributed to the expansion of group I/II members (Shinohara and Nishitani, 2021). Tandem duplication is one of the important mechanisms underlying the expansion of *XTH* family members, and tandem duplications comprise 4.56% of the genome of *L. chinense* (Chen et al., 2019). We detected a large number of tandemly duplicated *LcXTH* genes, accounting for 66.7% of all *LcXTH* genes, indicating that tandem duplication is the main mechanism underlying the expansion of *LcXTH* family genes. All the tandem repeats identified in this study were group I/II members, indicating that tandem duplication has been a particularly important mechanism underlying the expansion of group I/II members. Previous studies have shown that some gene family members have gained new functions or undergone defunctionalization (Lynch and Conery, 2000). In this study, tandemly duplicated genes on chromosome 17 were of particular interest. The expression patterns of *LcXTH13*, *19*, *20*, *21*, *22*, and *24* were similar, and their functions might have been retained after genome duplication. The expression patterns of *LcXTH16*, *18*, and *25* were inconsistent. The differential expression of these genes indicates that they might have gained new functions. *LcXTH01, 02*, *11*, and *17* were not expressed in any tissues in this study, suggesting that they might have undergone defunctionalization.

Understanding the classification of subfamilies is important for studying the evolution of the *XTH* gene family. Previous studies of monocotyledonous rice and dicotyledonous *A. thaliana* have shown that group I and group II members are difficult to distinguish; they have thus been usually classified into one group (group I/II) (Yokoyama et al., 2004). However, both sequence analysis and catalytic measurements have confirmed the divergence of group III (Fanutti et al., 1993; Yokoyama et al., 2004). In this study, a total of 27 *LcXTH* genes were identified, and they were grouped into three subfamilies. None of these genes were classified in the early diverging group, which might stem from an incomplete genome assembly or gene loss. Domain loop 2 in XTH family members plays an important role in subfamily classification, and previous studies have shown that the length of loop 2 contributes to the difference in the activity between XET and XEH members (Baumann et al., 2007). For example, the extension of loop 2 is thought to be the major structural change responsible for its endohydrolase activity in *Fragaria vesca* (Opazo et al., 2017). In this study, the extension of loop 2 was only observed in group III-A, and this might be an important factor contributing to the divergence of group III members. Glycosylation as one of key post-translational modifications can affect stability and the molecular weight of the target proteins (Ahmadizadeh et al., 2020; Heidari et al., 2020). In addition, proteins associated with the secretory pathway are firstly glycosylated in the endoplasmic reticulum. The above suggests that XET proteins may be involved in the secretory pathway. In this study, the N-glycosylation domain was observed in all LcXTH members, and previous research has demonstrated that the removal of this region significantly reduces the stability of some XET proteins (Campbell and Braam, 1999a; Kallas et al., 2005).

### The roles of *LcXTH* family members in abiotic stress and root development

Stomatal closure is one of the first events that takes place in plants in response to drought stress to prevent water evaporation, and *XTH* genes play a crucial role in modifying the cell wall and altering its elongation under drought stress, which improves drought resistance (Kim et al., 2010). For example, overexpression of *HvXTH1* in barley resulted in the enlargement of the stomata of transgenic plants relative to those of WT plants under drought treatment, indicating that transgenic plants were more drought tolerant (Fu, 2019). Overexpression of *XTH* genes from rose (*Rosa rugosa*) increased the drought tolerance of transgenic China rose plants (Chen et al., 2016). In addition, the presence of xyloglucan in early land plants suggests that *XTH* gene family members have played a key role in the transition from wetter to drier habitats (Popper, 2008). In our research, the drought treatment and RT-qPCR analysis showed that six *LcXTH* genes significantly responded to drought stress. The expression patterns of *LcXTH18*, *19*, *20*, and 25 were similar, suggesting that they exhibit similar responses to drought stress. ABA is an important signal in plants that mediates the response to drought stress. The most abundant phytohormone response element of *LcXTH* genes was associated with the ABA response (ABRE motif, 58.48%). Moreover, the augmented *NCED* expression levels detected in transgenic tobacco indicated that the increased drought stress resistance provided by the *LcXTH21* transgene probably involved the promotion of ABA biosynthesis.

The “balanced growth” hypothesis proposes that some plants can stimulate or maintain root growth while reducing shoot growth in response to drought stress (Bloom and Mooney, 1985). In *Eucalyptus globulus*, a drought-tolerant clone was found to have higher root growth rate than a drought-sensitive one (Costa et al., 2004). Moreover, the development of the root system largely determines the performance of plants under drought conditions. Thus, increased root biomass is one of the primary mechanisms used by plants to avoid, or reduce, drought stress (Kashiwagi et al., 2005). For example, the overexpression of *OsNAC5* was found to enhance drought tolerance by increasing root diameter (Jeong et al., 2013). *XTH* gene family members have been widely studied considering the different roles they are known to play in root development. For example, seven *XTH* genes from rice were specifically expressed in the roots of seedlings (Yokoyama et al., 2004). *AtXTH19* and *AtXTH23* were involved in lateral root development *via* the *BES1*-dependent pathway, indicating that *XTH* genes play a role in root development (Xu et al., 2020). Later, the expression levels of *AtXTH11*, *AtXTH29*, and *AtXTH33* in the roots and aboveground organs were found to differ in *A. thaliana* plants subjected to high temperature and drought stress, thereby suggesting that these genes might mediate rapid responses to drought stress (De Caroli et al., 2021). In a study performed in grapevine, the transcription levels of *VvXTH* genes presented the largest changes in roots and leaves under drought and salt stress, indicating that *VvXTH* genes vigorously respond to abiotic stress in leaves and roots (Qiao et al., 2022).

Molecular breeding is a promising approach and great progress has been made in its use for improving the efficiency of plant breeding programs (Varshney et al., 2009). Combined with molecular marker-assisted selection, greater and faster genetic progress can be achieved. For example, in a study of *Triticum aestivum*, Iquebal et al. (2019) found that the maximum number of drought responsive quantitative trait loci were detected at the seedling stage and further analyzed the regulatory networks of key candidate genes and their roles in responding to drought stress in order to identify putative markers for breeding applications. Besides, in a recent study, Chauhan et al. (2022) used morphological, biochemical, and molecular markers from *Withania somnifera* to assess the 25 accessions of Indian ginseng, and concluded that these markers could be used to select superior ginseng genotypes. In addition, *XTH* genes were also reported to be involved in other abiotic stress, such as salt, heat and cold stress (Han et al., 2017; Hidvégi et al., 2020; De Caroli et al., 2021). Hence, we suggest that *LcXTH* genes play roles in drought resistance and other abiotic stresses. In this study, we performed functional characterization of *LcXTH21*, and transgenic tobacco showed higher drought resistance and more developed roots during seedling stage. Therefore, *LcXTH* genes could be potential functional markers when conducting marker-assisted-selection (MAS) for breeding varieties with high resistance to abiotic stress. As an example, we could select genotypes with high *LcXTH21*expression levels and these MAS genotypes could be expected to have higher resistance to drought stress at the adult stage. This procedure could represent an alternative way to breed *L. chinense* varieties with increased stress resistance associated with a highly developed root system in the coming decade.

## Conclusion

In this study, 27 *LcXTH* genes were identified, and they were divided into three subfamilies. Tandem duplication was probably the major contributor to the expansion of the *LcXTH* family, and six *LcXTH* genes significantly responded to drought stress. Overexpression of *LcXTH21* in tobacco resulted in a more developed root system. In summary, these findings enhance our understanding of the *LcXTH* gene family and lay the foundation for further exploration on drought resistance mechanisms in *L. chinense*.

## Data availability statement

The original contributions presented in the study are publicly available. This data can be found here: NCBI, PRJNA559687.

## Author contributions

JW performed the experiments, analyzed the data and wrote the manuscript. YZ contributed to the data analysis and preparation of the manuscript. ZT took a role in experimental design and data analysis. ZH analyzed the data. LY, WL and ZC participated in plant sample collection and experimental assay. HL conceived the project, designed the experiments and revised the manuscript. All authors contributed to the article and approved the submitted version.

## Funding

This study was financially supported by the National Natural Science Foundation of China (31770718 and 31470660) and the Priority Academic Program Development of Jiangsu Higher Education Institutions (PAPD). The funding bodies played no role in the design of the study and collection, analysis, and interpretation of data and in writing the manuscript.

## Conflict of interest

The authors declare that the research was conducted in the absence of any commercial or financial relationships that could be construed as a potential conflict of interest.

## Publisher’s note

All claims expressed in this article are solely those of the authors and do not necessarily represent those of their affiliated organizations, or those of the publisher, the editors and the reviewers. Any product that may be evaluated in this article, or claim that may be made by its manufacturer, is not guaranteed or endorsed by the publisher.

## Supplementary material

Supplementary Figure 1 Different transgenic plant stages.

Supplementary Figure 2 Distribution and synteny of *LcXTH* genes in *L. chinense* chromosomes. Blue lines indicate synteny between *LcXTH* genes. Different colors indicate different chromosomes and syntenies. Gene densities are shown as red lines in the box. And the *LcXTH* genes were marked.

Supplementary Figure 3 The identity heat map of *LcXTH* genes.

Supplementary Table 1 The primer used in this study.

Supplementary Table 2 The characteristics and subcellular localization of LcXTH proteins.

Supplementary Table 3 The substitution rates (Ka/Ks) and diverging time of *LcXTH* gene pairs.

Supplementary Table 4 The protein similarity matrix of *LcXTH* genes.

Supplementary Table 5 The GO annotations of *LcXTH* genes.

File S1 The protein sequences used in phylogenetic tree constructing, and open reading frame sequence of *LcXTH21*.
