# Supplementary figures and images for: Genome-wide identification of XTH genes in Liriodendron chinense and functional characterization of LcXTH21
Source: Front Plant Sci. 2022 Oct 27;13:1014339. doi: 10.3389/fpls.2022.1014339 (PMC9647132; doi:10.3389/fpls.2022.1014339)

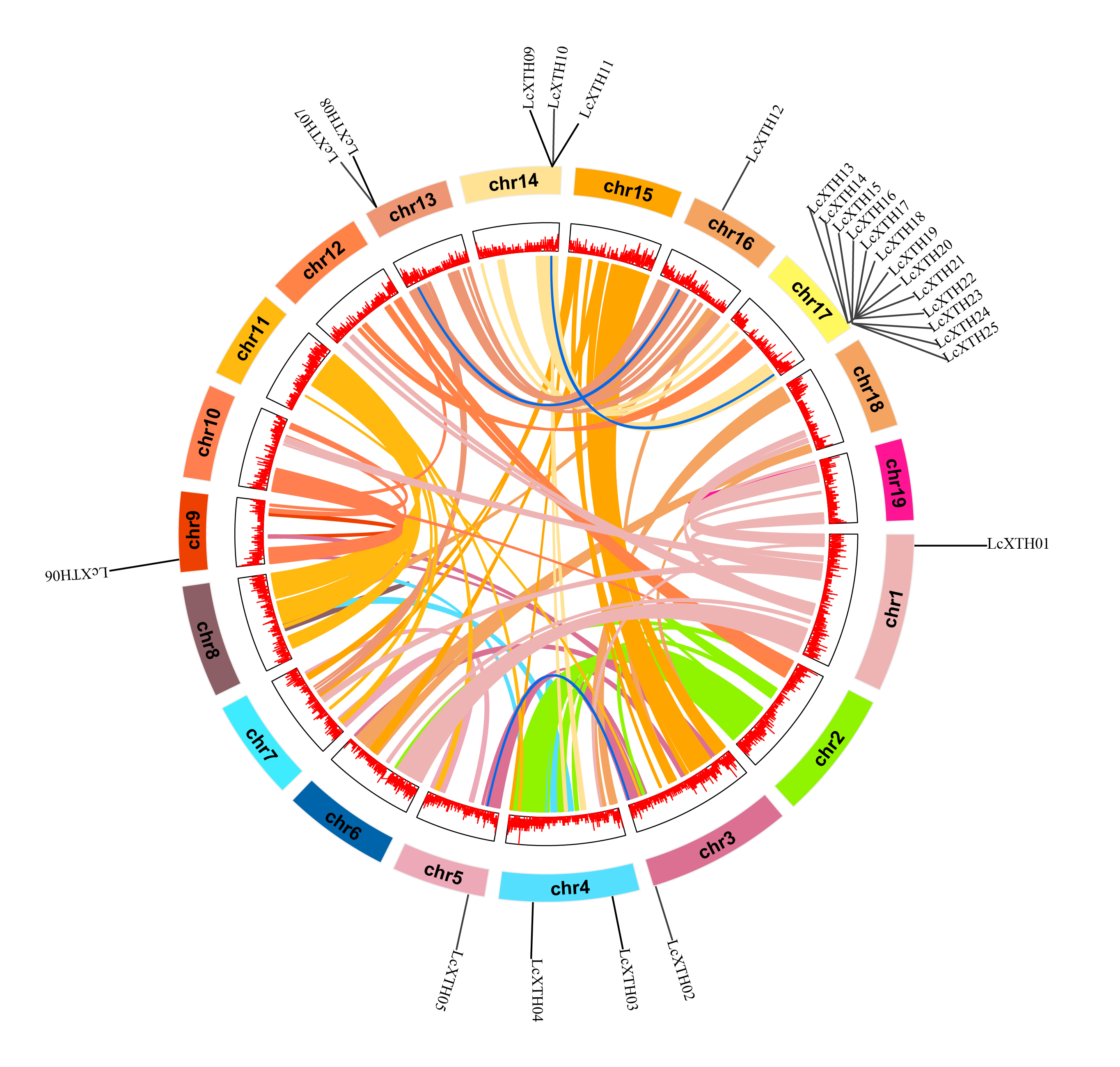

Supplement: Supplementary Figure 1 — Different transgenic plant stages. [file Image_1.jpeg]

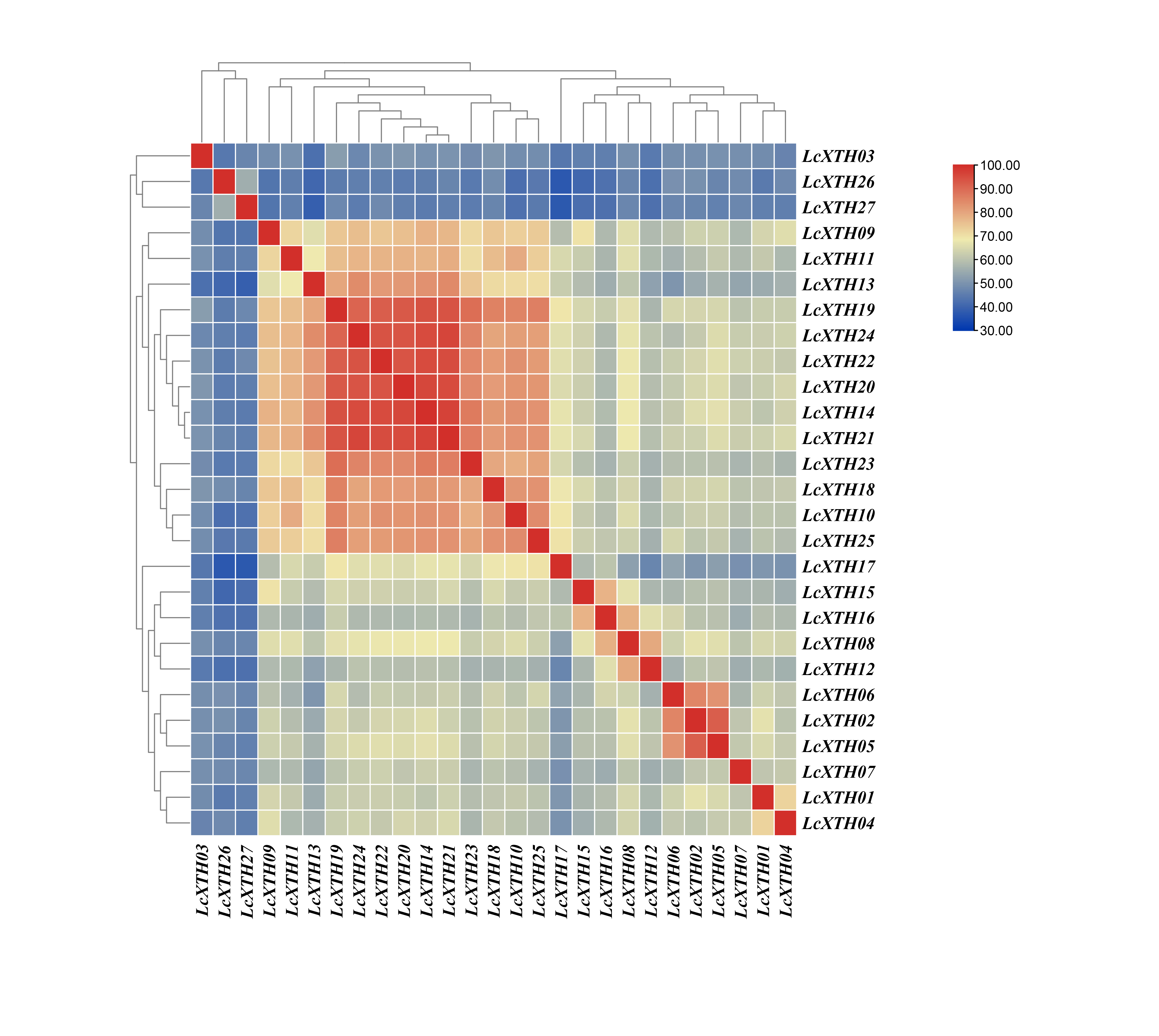

Supplement: Supplementary Figure 2 — Distribution and synteny of LcXTH genes in L. chinense chromosomes. Blue lines indicate synteny between LcXTH genes. Different colors indicate different chromosomes and syntenies. Gene densities are shown as red lines in the box. And the LcXTH genes were marked. [file Image_2.jpeg]

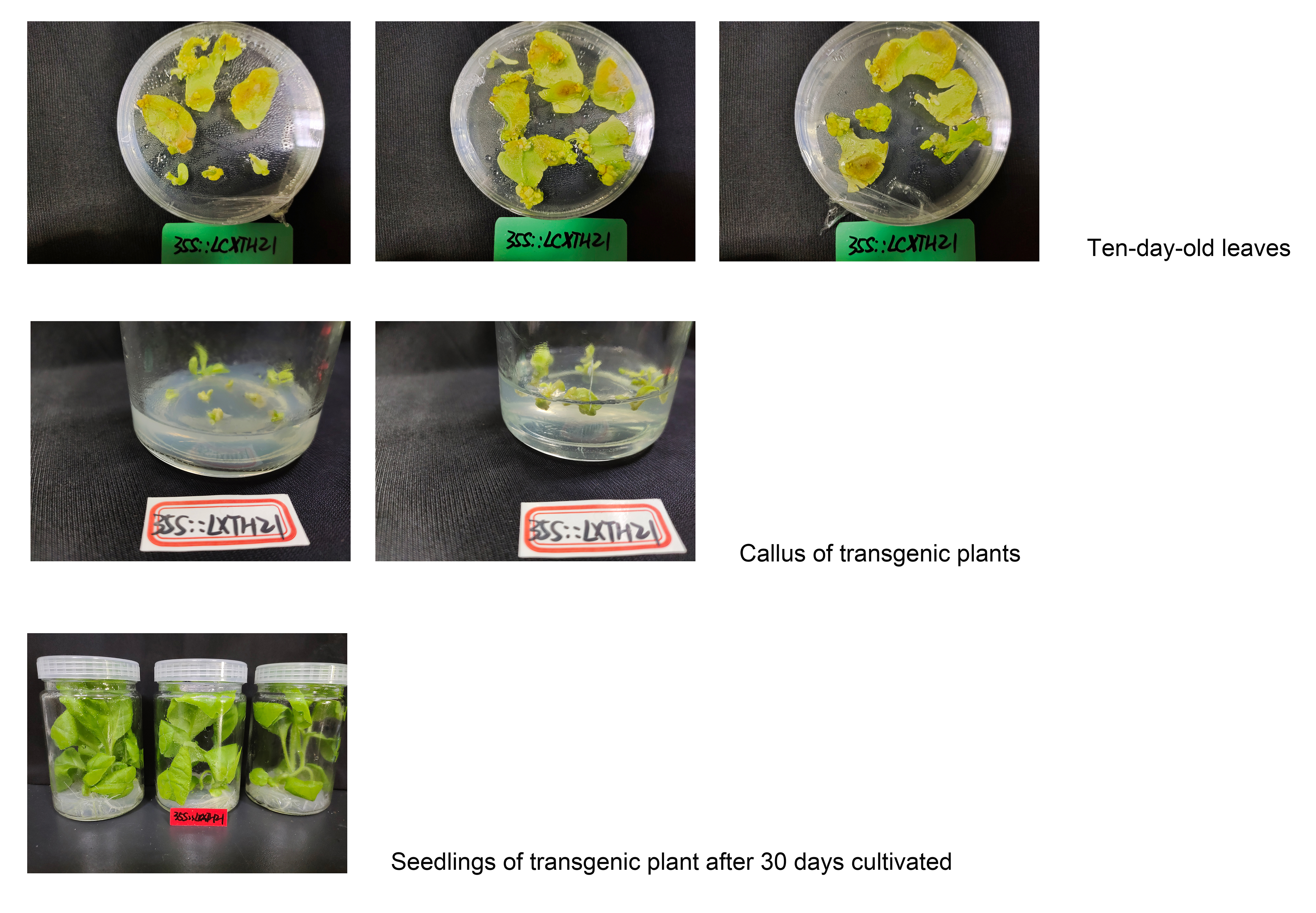

Supplement: Supplementary Figure 3 — The identity heat map of LcXTH genes. [file Image_3.jpeg]

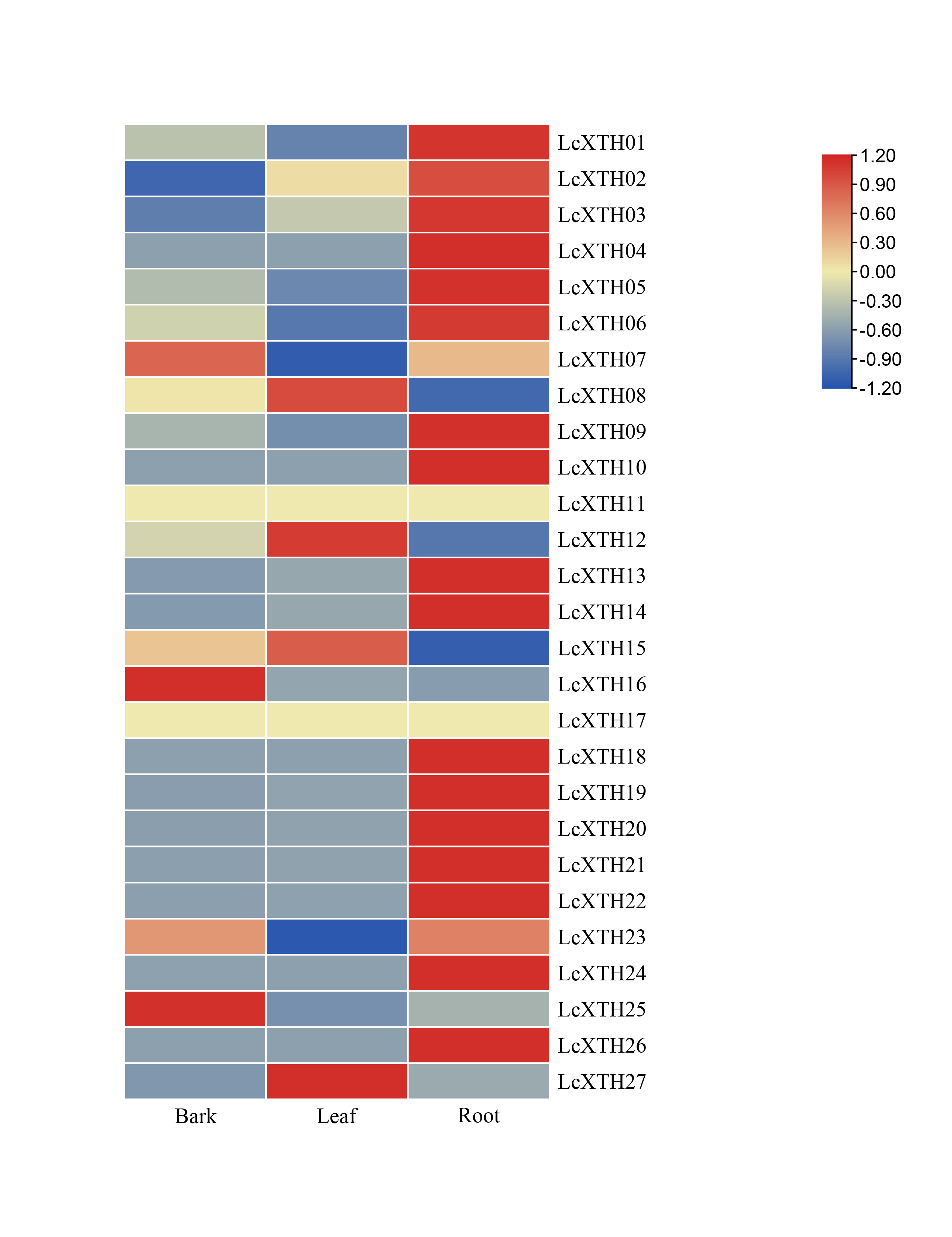

Supplement: Supplementary file 4 [file Image_4.jpeg]
