# Supplementary material for: Genome-wide identification of XTH genes in Liriodendron chinense and functional characterization of LcXTH21
Source: Front Plant Sci. 2022 Oct 27;13:1014339. doi: 10.3389/fpls.2022.1014339 (PMC9647132; doi:10.3389/fpls.2022.1014339)
Supplement: Supplementary Table 1 — The primer used in this study. [file Table_1.docx]

>OsXTH01

MGSLGRRPWVGGLTAAMIFAVAVCGFCFSGASAAAAAPTFGDNFEITGAEDHVKTSADGQTWYLYLDNKTGVGFQTKERYLFGWFSMNLKLAGNDSAGVVTAYYMCSDVDAAPQRDELDFEFLGNRTGEPYIIQTNVYRSGVGGREMRHSLWFDPTADFHSYSILWNPKQIVFFVDKVPIREYRNSDKPNTFFPIMKPMYVFSSIWNADDWATRGGLEKTDWTKAPFISSYRDFTADACSWGTAAASPPSCAASTGNSWWDQPPAWALDAGQREDSAWVARNLVIYDYCDDRKRFPSPPEECLLRTTSS

>OsXTH02

MATTTAAAMVVAMSVLLLGGGEAAAPRKPVDVAFEKNYVPTWAEDHIHYVDGGREVQLYLDKSTGTGFQTRGSYLFGHFSMHIKLVAGDSAGTVTAFYLSSQNSEHDEIDFEFLGNRTGEPYILQTNVFSGGKGDREQRIYLWFDPTKDYHSYSVLWNLYMIAFFVDDTPIRVFKNSKDLGVRYPFNQPMKLYSSLWNADDWATRGGREKTDWSRAPFVASYRGFHVDGCEASAEARYCATQGARWWDQPEFRDLDADQYRRLAWVRKTHTIYNYCDDRERYPAMSPECHRDRDA

>OsXTH03

MASLAVVVVVVAVVCAAGVAAAGKFDDVVEPSWANDHVVYEGDLLKLRLDSSSGGGFASKSKFLYGKATADLKLVAGDSAGVVTAFYLSSGGDKHNEFDFEFLGNVTGEPYLVQTNLYIDGVGNREQRIDLWFDPTADFHTYAVLWNPSQVVFLVDDTPIRVYENKNATAAVKGHHRHAAAANGTSNATSAAASVPPFPSPQPMSVYSSIWNADDWATQGGRVKTDWSHAPFVATFRDVRVEGCAWAANATDSDAGEVARCTGSSWGKEGRYWWKEKDMEELTVHQNHQLVWARAHHLVYDYCVDTDRFPVQPPECAGR

>OsXTH04

MGQARAHLLASLWAFYLILAISMVTGDLTNDLDILWGNSKVFYDNSGKQTISLTLDRWTTSAFRSKSTYLFSRIDMDIKLVAGDSAGTVTTLYVSGQGMNRLLILVDDKLIRQIKNNLMYSVPYPTYQPMRVYGSIWNADDWATMGGRVKTDWSQAPFTAYFRNYRAIACPPQQSSPLCGQSSGNWFNQELDVTRKQQLQEVDANYKIYDYCTDTKRFKDNLPKECTIN

>OsXTH05

MGRLANWLDDRTSKSTPDYRIQAGSFLLKLWFRPIFIIFFMPTSSCQPMMSSSYTFQESKHTPQSAYSLRHFALAKAMGQPRAQLLPSMSMAALYLILATSPVISDMTDSLDMLWGNTQVLYDSTGHQIVSLSLDRWTTSAFRSKTKYLFARIDMDIKLVAKDSAGTVTTLYMITEGLWDIHDEIDLEFLGNTTGEPYTLHTNIYARGTGGREKQYRLWFDPTEDFHTYTIIWNPQMILILVDGTPIRQMKNQLRNDIPFPLYQPMRLYASIWDADDWATQGGRIKTDWSQAPFTAFFRNYQANACIPYKTAWICSQGSNDSSWFTQDLDEEGKQKLKDVDDNYKIYDYCTDSRRYPNGYPPECGSQ

>OsXTH06

MAPARAHHLLACLLASALLAAATPVTGGGLMTDQLEVLFGQTQLLNDSNGDQTIALTLDREMGSAFKSKTSYLFARIDMDIKLVADDSAGTVTTIYLISEKDWNTHDEIDLEFLGNVTGQPYTLHTNIFANGEGGREVQYRLWFDPTQDFHTYSVIWNPDEILILVDNMPIRQFKNHLDSGVPFPIYQPMRLFGCLWDADDWATEGGRIKTDWSQAPFVAYFRNYTADGCVPSSYAWVCGQGPASSSDWFDRGLDDVKQQQQLREAQDKYMIYNYCNDPERFPDGYPKECGLQ

>OsXTH07

MAPMPLASSSSKLCSLLILCLAFLAAVDRSTAGIFDEIELIWGASRTYFFMDGDSEALALSLDQSQGSCFRSREKYLYVQIDVEIKLIEGDSAGTVCTIYTISEGPWEIHDEIDLEFLGNVTGEPYTLHTNIFANGVGGREQQFRLWFDPTADYHTYSIVWNPKRILILVDGKAIRDFKNNEDQGVPFPTWQSMRTFGSLWSAEDWATQGGRVKTDWKQAPFVTYYRNYNVTWCRPSPGVAWCGDEPKDSTRFDLDANTLSDLQWVRSNSMIYNYCDDSVRFNATTLPKECTLQ

>OsXTH08

MAMPCSGERCRRVWWSAAAAVVAFFFVVFVAAAAAAATASMYDDVEVVWGGDHSFFFMDGDGDALALCLDETHGSGFRSRDAYLYARFDVDMMLVANNSAGTVTTLYLMPDDVPWEYHDEVDLEFLGNVTGEPYTLHTNIFANGVGGREQQFRLWFDPTADFHTYSIVWNPKHIIILVDGVPIRDYRNTAARGGPAFPTWQKMRAHGSLWNADDWATQGGRVKTDWSEAPFFAYYRGLRVTPCAPSPGVAWCGDEPPESPWFDQQEMDAAALSKARQEHLLYDYCEDTKRFKDTGLPVECTIN

>OsXTH09

MGFGSREMACALVALVLGLCCVGGARATGRIDEGLEVMWGDGRGSVSPDGQVMALSLDHTSGSGWRSKNTYLFARVDLQIKLVANNSAGTVTTCYFMSEGEWDIHDEVDLEFLGNVTGQPYTLHTNVFANGTGGKEQQFHLWFDPTTDFHTYSIVWTSQHILVLVDGTPIREMKNHADKGIAYPSSQRMRLYGSLWNADDWATQGGRVKTDWSQAPFVARYRNFTATEAASSSSPAGYDQQMDATAQQAMKWARDNYMVYDYCADSKRFPQGFPPECSMP

>OsXTH10

MAALLVVVLVAMSAMVATANFNQEFDITWGDGRGKILEDGQLLTLTLDRTSGSGFQSKHEYLYGKIDMQLKLVPGNSAGTVTAYYLSSQGPTHDEIDFEFLGNVTGEPYTLHTNVFTQGQGQREMQFRLWYDPTKDFHTYSILWNPKHIIFMVDDMPIRDFRNLEGKGIAFPKNQPMRLYSSLWNADDWATQGGRVKTDWTHAPFSASYRGFRADACVVAAGGRTRCGATVGTDAAPGTGAAAAAGGWYNQELDLTRQQRMRWVQSKYMIYNYCTDPKRFPQGVPAECSM

>OsXTH11

MRTVALGIVAMACLVAIAHGGNFFQDAEVSWGQGRGKIVDGGRGLDLTLDRSSGSGFQSKSEYLFGKIDMQIKLVPGNSAGTVTTFYLSSQGSTHDEIDFEFLGNVTGEPYTLHTNVFTQGQGQREQQFRLWFDPTQSFHTYSIIWNPQHVIFAVDGTPIRDFKNHEARGVAFPKSQPMRVYASLWNADDWATQGGRVKADWSKAPFVASFRDFNADACVWSNGAQRCPVGTMETVAAPAGGRRGGAGGWWNQELSDMSYRRMRWVQRKFMIYNYCTDAKRFPQGTPAECKLR

>OsXTH12

MVSSSNPGRTPPLVAAIVCSVLLLAGGAAGNFYQDVDITWGDGRGKILGNGQLLTLSLDRSSGSGFQSKNQYLYGRFDMQIKLVPGNSAGTVATFYLSSQGSQHDEIDFEFLGNASGEPYTVHTNVYSQGKGGREQQFRMWFDPTKDFHTYSVLWNPSHILFYVDGTPIREYRNTEATTGVAFPRAQAMRVYASLWDAEEWATQGGRVRTDWSRAPFTASYRGLAASGCTSQDATACANPGSPWMYQQQLDSASQDRLRQVQRDYMIYNYCADTYRFPQGLPPECTAK

>OsXTH13

MAAKLQGGGGGAAVMAVVVVAMVAGAASGGNFYEECDATWEPQNCWSSDNGKSLSLALVSNSSGSMIRSKRQFVYGSVSTSVQLVPGNSAGTVTTFYTSSLGDKHDEIDFEFLGNETGQPYTIHTNVYANGVGDKEMQFKPWFDPTDGSHNYTISWTPCRIVWYIDGMPIRVFRNYQSSNGVAFPTWQPMYAYSSIWAAEDWATQKGRVKTDWSKAPFVANYHGIDLDVCECYGGDCVYGCAAAFNQGGGCAGQQLTGDEMGQMKWVQDNFRIYDYCVDYKRFNGQMAPECSLPQY

>OsXTH14

MGRLSLLLVVFTAAAAVVGLAGASFRDECDIPWEPQNARFTDDGNGLSLSLVSNYSGCMLRTKKQFIFGSVSTLIQLVPGNSAGTVTTYYTSSVGDNHDEIDFEFLGNETGQPYTIHTNIYANGVGDKEMQFKPWFNPTDGYHNYTVSWTACMIVWYIDGTPIRVFRNYEKSNGVAFPMKRPMYGYSSIWAAEDWATQGGRVKADWSKAPFVANYHGLNINVCECSTTSGGGNSCAAKCASTYNSKSSVCQLSDSELARMRKVQDEYRIYNYCVDPKRYNGSVPVECSLPQ

>OsXTH15

MAKALLAVVVVAVAAVLELGLVGANFQDQCDITWEPQNAKMTEGGDHLTLSLVSNSSGCMLRTKKQFIYGSVSTRIQLVKGNSAGTVTTYYTSSIGDKHDEIDFEFLGNSSGLPYTFHTNVFADGVGSREMQFRPWFDPTDGYHNYTIFWNPCMIVSHWTDDSIERHKYFCFRWFVDSIPIRVFRNHEKEGVPFPTKRPMYAFSSIWAAEDWATQGGRVKTDWTKAPFVAEYRDIGLNICECPGSGSGSSSSFSSSSSSTSGDAEDPACAQRCATSDHWYAAEGLCQLSDKQLRQMKAVQLGYTIYDYCADAQAKGRPVPPECSMPQY

>OsXTH16

MARPLGQQQVGAAAALVIVVACCVVAGCSGARGRGFREEFDVIWGEDHVRVTDEDDAATRQVVALTLDQASGSGFQSKDQFLFGEFSMEMKLVPGESPGTVATFYLTSEGDAHDEIDFEFLGNVSGEPYVMHTNVFAQGRGNREQQFYLWFDPTADFHNYTILWNPLNIIFSVDGKAVRVFKNHEAAGVPYPSGQAMRVHASLWNGDFWATRGGQVKINWTAAPFVASYRTYAYSACAVPAAGGGGGGPCTSGQLPNSTSSPSTCDCGGAWMDRQLGADGERDVAWARANYMIYDYCGDQWRFPQGLPAECSLDQSSGHRT

>OsXTH17

MAKHLALSVAAAVAVSWLAASSAAAAGFYEKFDVVGAGDHVRVVSDDGKTQQVALTLDRSSGSGFTSKDTYLFGEFSVQMKLVGGNSAGTVTSFYLSSGEGDGHDEIDIEFMGNLSGNPYVMNTNVWANGDGKKEHQFYLWFDPTADFHTYKIIWNPQNIIFQVDDVPVRTFKKYDDLAYPQSKPMRLHATLWDGSYWATRHGDVKIDWSGAPFVVSYRGYSTNACVNNNPAGGWSSSWCPEGTSAWIHRELDGAELGTVAWAERNYMSYNYCADGWRFPQGFPAECYRK

>OsXTH18

MRGGASLRLRWPAALVAVVAAAVTAAAAAGHGDHNFHRDFDAVWGKGNARFRDGGRMVELTLDEQTGARLQSKERFLFGRFDLEIKLVRGESAGTITSFYICSGGARHDEVDFEFLGNVSGEPYLLHTNIFSDGKGEREQQFVLWFDPTADFHTYSILWNPHNIILYIDGTPIRVFKNNEAYGVPFPTRQPVHVFASIWNAEEWATQGGRVKTDWSRAPFVATYRRYNVSNACVWDAAGAGASRCAGGGGGWMRRRMDWWSWMTLNWVRMNYMAYDYCADRKRFPHRFPAECIIPIGRT

>OsXTH19

MEQKPPAVAANNNQLLLMMIMVVVACSNYMISGAGAQPSPGYYPSKTIRSMAFGEGYDNLWGGQHQTLSADQTALTVWMDRSSGSGFKSKRSYRNGYFGASIKVPSGYTAGVNTAFYLSNNELYPGQHDEIDIELLGTVPGEPWTLQTNVYVHGTGDGAIIGREMRFHLWFDPTADFHHYAILWNPDHIVFLVDDVPVRRYPRAAGNTFPDRQMWAYGSIWDASDWATDGGRYKSDYRYQPFVSRYRDLKIAGCEAAAPASCQPVPASPSGATGELSAQQKAAMRWAQQRSMVYYYCQDYSRNHANYPEC

>OsXTH20

MARPGSGNIPGSACIPLLILLLLLLLLHPSEAQPSPGYYPSKMFRSMAFYEGYSTLWGPQHQTLSQDQKSLTLWMDRSSGSGFKSTRSYRNGYFGASIRVQPGYTAGVNTAFYLSNTEQYPGHHDEIDMELLGTVPGEPYTLQTNVYVRGSGDGNIVGREMRFHLWFDPTAGFHHYAILWNPDQILFLVDDVPIRRYEKKVEGTFPEREMWAYGSIWDASDWATDGGRYRADYRYQPFVSRFADLKVGGCATAAPPACSPVPASSGGGSAALSPQQEAAMAWAQRNAMVYYYCQDYSRDHTFYPEC

>OsXTH21

MAASAAAPATVAGLLVAVAAIMAASPAGAQPSPGYYPSSVHRAMAFSRDYTNKWGPQHQTLSADQSSLTIWLDKTCGSGFKSRKSYRNGYFAARVKLPAGYTAGTNTAFYLSNNEAHPGFHDEIDMEFLGTIPGEPYTLQTNVYVRGSGDGRIVGREMRFHLWFDPTADFHHYAILWNPDAITFFVDDVPIRRYERKSELTFPDRPMWVYGSIWDASDWATDDGRHRADYRYQPFVARFDRFTVAGCAPSAPASCRPVPASPAGAGLTPRQYAAMRWAQQSHMVYYYCQDYRRDHSLTPEC

>OsXTH22

MAIIGRRQQQGVAAAAATLVALMAVVVAAAAEAQPSPGVYPSRMFRAREFGRDFRSLWGAEHQQQEAAAPETGVTVWLDRRSGSGFKSRRAYRSGYFGAWVRLQRGYTAGVITAFYLSNGEAHPGWHDEVDMEFLGTTPGKPYTLQTNVFSLGSGDPPRSLGREIKFHLWFDPTADFHHYAILWTSDHIIFLVDDVPIRRYGRRSAGGAAGFPARPMWVYGSIWDASSWATEDGRYRADYSYQPFVARFSAFLLRGCSPHAPRTCAAPVAGDLTAAQLAAMRWAQRFHMVYNYCYDPKRDHSLTPECRTHLHPSSSSSNSSSSSYHG

>OsXTH23

MALEARFFLAAVFAVAATCLCLSAVASAFAVPSVAFDEGYSPLFGDDNLVRSSDDKSVRLLLDRRSGSGFISSDYYLHGFFSASIKLPKAYTAGVVVAFYLSNGDVYEKTHDELDFEFLGSRWGGQWRVQTNAYGNGSTARGREERYLLPFDPTLEAHRYSVLWAPTHIIFYIDDTPIREVIRHPGMGGDFPSKPMAVYATIWDGSTWATDGGKYKVNYKYAPFASEFSDLALLGCRADPVLRAPRDGGGAGCAEPDLLGLLTADYAVMTPRKRAAMRAFRARHMTYTVCYDAVRYAAGPFPECDVSDVEKESFSAWGESKNVVMKARGRGRRRGRKAGAGAMSRLDVSSS

>OsXTH24

MAAAVLAVLWACMMMMSLAPASLAASGFEEVPTIAFDEGFSPLFGEDNMVKSADGRTVSITLNRYTGSGFISSDYYHHGFFSASIKLPKDHTAGVVVAFYLSNGDVFEKTHDELDFEFLGNRYRHEWKMQTNVYGNGSTDRGREERYLMPFDPTADAHRFSILWHSRLIVFYVDGVPIREVPRTAAMGADYPSKPMALYVTIWDGSTWATDNGKYKVNYKRGPFTAVFSDLVLRGCTARSDIRLATTADDQDRCAAAEEDLMESDEYSSTMAMTARKRMAMRRFRQRQMLYTVCYDTNRYPEPFPECDVNMAERQMYWQWGESKVVRPRVRPRPGRRSKRRPSPEATAIPPPVLVSLQQAD

>OsXTH25

MTTSSWSGLLVISCMLLMSWAAAAVDMSPVRFDAAYMPLFGGDNLVPSPHARTVLLKLDRFTGSGFVSKSAYHHGFFSASIKLPHDYTAGVVVAFYLSNGDVFPGQHDELDFELLGNRRGHAWHVQTNMYGNGSTGRGREERYLLPFDPTAAPHSYAIAWTPAAVIFYIDAIPIRELVRCSSGDYPAKPMSVYATIWDGSAWATDGGRHKVDYAYAPFTAVFSDLVVTGGTDDDHCAAMGLMTSEVAVMTPAKRGSMRRFRSRHLTYSACYDTVRYNGTGVVFPECDESEQDNFHAWGESKRVINSRSSSSATYATGSGVRID

>OsXTH26

MAGRRLLVATAVVAAAAAVVAAAALEAINVTTVAFEEGYTPLFGFDNILRSADDRTVSLLLDRSTGSGFMSSSMYQHGFFSASIKLPSDYTAGVVVAFYTSNGDVIEKRHDELDFEFLGNIRGKPWRVQTNVYGNGSVSRGREERYLLPFDPTTEFHRYSILWTRAAIVFFVDDVPIREVRRTPAMTGDFPSKPMSIYATVWDASTWATSGGRYRVNYRYGPFVASFTDLALLGCRVGDPIGQMLSSAACTAAEDALLASDLAVMTLEKQQAMRRFREQNMVYSYCYDTLRYPAPFLECDVVESERRRFKGSGHLRLAFRRRRRTRPGSRPARPTRAADM

>OsXTH27

MAASRCFLLLLLLLLSPLLASAGEEEEEAVLAMAARLRRPAAASFREGYTQLFGDSNLALHGDGKRVRISLDERTGAGFASQDAYLHGFFSASIKLPPDYAAGVVVAFYMSNGDVYEKTHDELDFEFLGNIKGREWRVQTNVYGNGSTSVGREERYGLWFDPTEDFHRYAILWSHDWIVFYIDETPIREVQRTKSMGVQFPSKPMSLYATIWDGSSWATSGGRYKVNYKYAPFVAEFSELMLHGCAMDTLTRAPMCTPDIANIHNAVAMSGRQRSAMERFRTKYMTYGYCYDRLRYPTPPSECNVGPEAELFLPTGEARSIDRHGRARRHRRGPADSAF

>OsXTH28

MAMARCSLLPILAAVLLAASLSLPPRAAAYAAMVDSLLPASATALSFEEGYTQLFGDSNLMLHGDGKRVHISLDERTGAGFASQGAYHHGFFSASIKLPADHTAGVVVAFYMSNGDVYERTHDELDFEFLGNVRGREWRVQTNVYGNGSTAAGREERYGLWFDPTQDFHRYAIRWSHDTIIFYVDETPIREVVRTASMGAQFPSKPMSLYATIWDGSSWATSGGRYKVNYKYAPYVAEFTDLLLHGCPAGSPPPCEGAAASATMPPGQRSAMERFRARHMTYGYCYDRVRYHAPLPECSVGAEAEAFLPSGEARSTDRRGGRHGKRHRRAGGGVDSAL

>OsXTH29

MVVVVAMPPALSLLVLLVLALHGGAGDATPPPPLRLVRGARRVAFDEGYTRMFGDGNLAVLRDGRRVRLTLDESTGAGFASQDVFLHGFFSAAVKLPAYYAAGVVVAFYLSNGDTYEKTHDEVDFEFLGNVRGREWRVQTNVYGNGSTAAGREERYDLPFDPTDELHHYSILWTRRRIIFYVDETPIREVVRTAAMGAAFPAKPMSVYATIWDGSAWATLGGRYRVNYRYAPFVAEFADLVLHGCAVDPLAVEHSASCGDEEEEAAEAVVSSAAMAAFRRGHMSYSYCHDRRRYPVALSECALTGGAASLGRLFGPDGMKRRRARRARDASS

>AtXTH33

MKIMWETAVVFCLCSLSLVSSHSRKFTTPNVTRLTDQFSKIAIENGFSRRFGAHNIQVNGSLAKLTLDKSSGAGLVSKNKYHYGFFSARLKLPAGFASGVVVAFYLSNAETYPKSHDEIDIELLGRSRRDDWTIQTNVYANGSTRTGREEKFYFWFDPTQAFHDYTLIWNSHHTVFLVDNIPVRQFPNRGAFTSAYPSKPMSLYVTVWDGSEWATKGGKYPVNYKYAPFVVSVADVELSGCSVNNGSSTGSGPCTKSGGSISSLDPVDGQDFATLSKNQINAMDWARRKLMFYSYCSDKPRYKVMPAECN

>AtXTH08

METERRIITSCSAMTALFLFMTALMASSSIAATPTQSFEDNFNIMWSENHFTTSDDGEIWNLSLDNDTGCGFQTKHMYRFGWFSMKLKLVGGDSAGVVTAYYMCSENGAGPERDEIDFEFLGNRTGQPYIIQTNVYKNGTGNREMRHSLWFDPTKDYHTYSILWNNHQLVFFVDRVPIRVYKNSDKVPNNDFFPNQKPMYLFSSIWNADDWATRGGLEKTDWKKAPFVSSYKDFAVEGCRWKDPFPACVSTTTENWWDQYDAWHLSKTQKMDYAWVQRNLVVYDYCKDSERFPTLPWECSISPWA

>AtXTH28

MGFITRFLVFMSLFTSLVSGFALQKLPLIQFDEGYTQLFGDQNLIVHRDGKSVRLTLDERTGSGFVSNDIYLHGFFSSSIKLPADYSAGVVIAFYLSNGDLYEKNHDEIDFEFLGNIRGREWRIQTNIYGNGSTHLGREERYNLWFDPTEDFHQYSILWSLSHIIFYVDNVPIREVKRTASMGGDFPAKPMSLYSTIWDGSKWATDGGKYGVNYKYAPYVSQFTDLILHGCAVDPTEKFPSCKDEAVQNLRLASEITESQRNKMEIFRQKHMTYSYCYDHMRYKVVLSECVVNPAEAKRLRVYDPVTFGGIPHGHRRGKHRSRSRLARTESI

>AtXTH30

MSKSSYNHIFILILCLCLRSSSAFTNLNTLSFEESLSPLFGDANLVRSPDDLSVRLLLDRYTGSGFISSNMYQHGFYSSMIKLPADYTAGVVVAFYTSNGDVFEKTHDELDIEFLGNIKGKPWRFQTNLYGNGSTHRGREERYRLWFDPSKEFHRYSILWTPHKIIFWVDDVPIREVIRNDAMGADYPAKPMALYATIWDASDWATSGGKYKANYKFAPFVAEFKSFSLDGCSVDPIQEVPMDCSDSVDFLESQDYSSINSHQRAAMRRFRQRFMYYSYCYDTLRYPEPLPECVIVPAEKDRFKETGRLKFGGTEARERRRNRRQQRRPEIEIESDPDDRKLL

>AtXTH17

MKLSCGTSFAFLLLFLLAAQSVHVYAGSFHKDVQIHWGDGRGKIHDRDGKLLSLSLDKSSGSGFQSNQEFLYGKAEVQMKLVPGNSAGTVTTFYLKSPGTTWDEIDFEFLGNISGHPYTLHTNVYTKGTGDKEQQFHLWFDPTVNFHTYCITWNPQRIIFTVDGIPIREFKNPEAIGVPFPTRQPMRLYASLWEAEHWATRGGLEKTDWSKAPFTAFYRNYNVDGCVWANGKSSCSANSPWFTQKLDSNGQTRMKGVQSKYMIYNYCTDKRRFPRGVPAECT

>AtXTH27

METLSRLLVFMSLFSGLVSGFALQNLPITSFEESYTQLFGDKNLFVHQDGKSVRLTLDERTGSGFVSNDYYLHGFFSASIKLPSDYTAGVVVAFYMSNGDMYEKNHDEIDFEFLGNIREKEWRVQTNIYGNGSTHSGREERYNLWFDPTEDFHQYSILWSDSHIIFFVDNVPIREVKRTAEMGGHFPSKPMSLYTTIWDGSKWATNGGKYGVNYKYAPYIARFSDLVLHGCPVDPIEQFPRCDEGAAEDMRAAQEITPSQRSKMDVFRRRLMTYSYCYDRARYNVALSECVVNPAEAQRLRVYDPVRFGGIPRRHRNGKHRSKRSRVDGTESI

>AtXTH04

MTVSSSPWALMALFLMVSSTMVMAIPPRKAIDVPFGRNYVPTWAFDHQKQFNGGSELQLILDKYTGTGFQSKGSYLFGHFSMHIKLPAGDTAGVVTAFYLSSTNNEHDEIDFEFLGNRTGQPAILQTNVFTGGKGNREQRIYLWFDPSKAYHTYSILWNMYQIVFFVDNIPIRTFKNAKDLGVRFPFNQPMKLYSSLWNADDWATRGGLEKTNWANAPFVASYKGFHIDGCQASVEAKYCATQGRMWWDQKEFRDLDAEQWRRLKWVRMKWTIYNYCTDRTRFPVMPAECKRDRDA

>AtXTH10

MTLINRSKPFVLLVGFSIISSLLLWVSQASVVSSGDFNKDFFVTWSPTHVNTSNDGRSRTLKLDQESGASFSSIQTFLFGQIDMKIKLIRGSSQGTVVAYYMSSDQPNRDEIDFEFLGNVNGQPYILQTNVYAEGLDNREERIHLWFDPAKDFHTYSILWNIHQIVFMVDQIPIRLYRNHGEKGVAYPRLQPMSVQASLWNGESWATRGGHDKIDWSKGPFVASFGDYKIDACIWIGNTSFCNGESTENWWNKNEFSSLTRVQKRWFKWVRKYHLIYDYCQDYGRFNNKLPKECSLPKY

>AtXTH21

MVSSTLLVMSISLFLGLSILLVVHGKDFNQDIDITWGDGRGNILNNGTLLNLGLDQSSGSGFQSKAEYLYGKVDMQIKLVPGNSAGTVTTFYLKSQGLTWDEIDFEFLGNVSGDPYIVHTNVYTQGKGDREQQFYLWFDPTAAFHNYSILWNPSHIVFYIDGKPIREFKNLEVLGVAYPKNQPMRMYGSLWNADDWATRGGLVKTNWSQGPFVASFMNYNSENACVWSIVNGTTTTSPCSPGDSTSSSSSSTSEWFSQRGMDSSSKKVLRWVQRKFMVYNYCKDKKRFSNGLPVECTAKNKNTKS

>AtXTH32

MGNSLISLLSIFHLLVLWGSSVNAYWPPSPGYWPSSKVGSLNFYKGFRNLWGPQHQRMDQNALTIWLDRTSGSGFKSVKPFRSGYFGANIKLQPGYTAGVITSLYLSNNEAHPGFHDEVDIEFLGTTFGKPYTLQTNVYIRGSGDGKIIGREMKFRLWFDPTKDFHHYAILWSPREIIFLVDDIPIRRYPKKSASTFPLRPMWLYGSIWDASSWATEDGKYKADYKYQPFTAKYTNFKALGCTAYSSARCYPLSASPYRSGGLTRQQHQAMRWVQTHSMVYNYCKDYKRDHSLTPECWR

>AtXTH16

MGRILNRTVLMTLLVVTMAGTAFSGSFNEEFDLTWGEHRGKIFSGGKMLSLSLDRVSGSGFKSKKEYLFGRIDMQLKLVAGNSAGTVTAYYLSSEGPTHDEIDFEFLGNETGKPYVLHTNVFAQGKGNREQQFYLWFDPTKNFHTYSLVWRPQHIIFMVDNVPIRVFNNAEQLGVPFPKNQPMKIYSSLWNADDWATRGGLVKTDWSKAPFTAYYRGFNAAACTVSSGSSFCDPKFKSSFTNGESQVANELNAYGRRRLRWVQKYFMIYDYCSDLKRFPQGFPPECRKSRV

>AtXTH03

MDYMRIFSVFVVTLWIIRVDARVFGGRGIEKFVTFGQNYIVTWGQSHVSTLHSGEEVDLYMDQSSGGGFESKDAYGSGLFEMRIKVPSGNTGGIVTAFYLTSKGGGHDEIDFEFLGNNNGKPVTLQTNLFLNGEGNREERFLLWFNPTKHYHTYGLLWNPYQIVFYVDNIPIRVYKNENGVSYPSKPMQVEASLWNGDDWATDGGRTKVNWSYSPFIAHFRDFALSGCNIDGRSNNVGACESSNYWWNAGNYQRLSGNEQKLYEHVRSKYMNYDYCTDRSKYQTPPRECY

>AtXTH31

MALSLIFLALLVLCPSSGHSQRSPSPGYYPSSRVPTSPFDREFRTLWGSQHQRREQDVVTLWLDKSTGSGFKSLRPYRSGYFGASIKLQPGFTAGVDTSLYLSNNQEHPGDHDEVDIEFLGTTPGKPYSLQTNVFVRGSGDRNVIGREMKFTLWFDPTQDFHHYAILWNPNQIVFFVDDVPIRTYNRKNEAIFPTRPMWVYGSIWDASDWATENGRIKADYRYQPFVAKYKNFKLAGCTADSSSSCRPPSPAPMRNRGLSRQQMAALTWAQRNFLVYNYCHDPKRDHTQTPEC

>AtXTH11

MRGSDQKILLMVMVVVAVVAAAQGQEETTGFVTWGNNYYQTWGHQALVINKTSELQLTLDKNSGSGFESQLIYGSGYFNVRIKAPQTTSTGVITSFYLISRSSRHDELCFQILGKNGPPYLLNTNMYLYGEGGKDQRFRLWFDPTKDYHSYSFLWNPNQLVFYVDDTPIRVYSKNPDVYYPSVQTMFLMGSVQNGSIIDPKQMPYIAKFQASKIEGCKTEFMGIDKCTDPKFWWNRKQLSSKEKTLYLNARKTYLDYDYCSDRQRYPKVPQECGSYT

>AtXTH09

MVGMDLFKCVMMIMVLVVSCGEAVSGAKFDELYRSSWAMDHCVNEGEVTKLKLDNYSGAGFESRSKYLFGKVSIQIKLVEGDSAGTVTAFYMSSDGPNHNEFDFEFLGNTTGEPYIVQTNIYVNGVGNREQRLNLWFDPTTEFHTYSILWSKRSVVFMVDETPIRVQKNLEEKGIPFAKDQAMGVYSSIWNADDWATQGGLVKTDWSHAPFVASYKEFQIDACEIPTTTDLSKCNGDQKFWWDEPTVSELSLHQNHQLIWVRANHMIYDYCFDATRFPVTPLECQHHRHL

>AtXTH01

MEYLSIFGFVSVLYLIIRVDARAYEVNGIDQSKVGFDDNYVVTWGQNNVLKLNQGKEVQLSLDHSSGSGFESKNHYESGFFQIRIKVPPKDTSGVVTAFYLTSKGNTHDEVDFEFLGNKEGKLAVQTNVFTNGKGNREQKLALWFDPSKDFHTYAILWNPYQIVLYVDNIPVRVFKNTTSQGMNYPSKPMQVVVSLWNGENWATDGGKSKINWSLAPFKANFQGFNNSGCFTNAEKNACGSSAYWWNTGSYSKLSDSEQKAYTNVRQKYMNYDYCSDKVRFHVPPSECKWNN

>AtXTH02

MNRIRYCFELVSVLFLMFTANARARGRGAIDFDVNYVVTWGQDHILKLNQGKEVQLSMDYSSGSGFESKSHYGSGFFQMRIKLPPRDSAGVVTAFYLTSKGDTHDEVDFEFLGNRQGKPIAIQTNVFSNGQGGREQKFVPWFDPTTSFHTYGILWNPYQIVFYVDKVPIRVFKNIKKSGVNYPSKPMQLVASLWNGENWATSGGKEKINWAYAPFKAQYQGFSDHGCHVNGQSNNANVCGSTRYWWNTRTYSQLSANEQKVMENVRAKYMTYDYCSDRPRYPVPPSECRWNQ

>AtXTH15

MGPSSSLTTIVATVLLVTLFGSAYASNFFDEFDLTWGDHRGKIFNGGNMLSLSLDQVSGSGFKSKKEYLFGRIDMQLKLVAGNSAGTVTAYYLSSQGATHDEIDFEFLGNETGKPYVLHTNVFAQGKGDREQQFYLWFDPTKNFHTYSIVWRPQHIIFLVDNLPIRVFNNAEKLGVPFPKSQPMRIYSSLWNADDWATRGGLVKTDWSKAPFTAYYRGFNAAACTASSGCDPKFKSSFGDGKLQVATELNAYGRRRLRWVQKYFMIYNYCSDLKRFPRGFPPECKKSRV

>AtXTH29

MRDSIYLLWIDNRLVVIIMMVMMVSCRCVLGLENINPIFFDEGLSHLFGEGNLIRSPDDRSVRLLLDKYTGSGFISSSMYQHGFFSSLIKLPGAYTAGIVVAFYTSNGDVFVKDHDELDIEFLGNLEGKPWRFQTNMYGNGSTNRGREERYRLWFDPSKEFHRYSILWTPTKIIFWVDDVPIREILRKEEMNGDYPQKPMSLYATIWDASSWATSGGKFGVDYTFSPFVSEFKDIALDGCNVSDSFPGENNNNNIGNYNNINCSVSDQFLMSNDYSTISPKQATAMRRFRERYMYYSYCYDTIRYSVPPPECVIVTAEKNRFRDTGRLKFGGSHPKVHKARKKRRRNRSTPVVSADL

>AtXTH23

MAMISYSTIVVALLASFMICSVSANFQRDVEITWGDGRGQITNNGDLLTLSLDKASGSGFQSKNEYLFGKIDMQIKLVAGNSAGTVTAYYLKSPGSTWDEIDFEFLGNLSGDPYTLHTNVFTQGKGDREQQFKLWFDPTSDFHTYSILWNPQRIIFSVDGTPIREFKNMESQGTLFPKNQPMRMYSSLWNAEEWATRGGLVKTDWSKAPFTASYRGFNEEACVVINGQSSCPNVSGQGSTGSWLSQELDSTGQEQMRWVQNNYMIYNYCTDAKRFPQGLPRECLAA

>AtXTH14

MACFATKQPLLLSLLLAIGFFVVAASAGNFYESFDITWGNGRANIFENGQLLTCTLDKVSGSGFQSKKEYLFGKIDMKLKLVAGNSAGTVTAYYLSSKGTAWDEIDFEFLGNRTGHPYTIHTNVFTGGKGDREMQFRLWFDPTADFHTYTVHWNPVNIIFLVDGIPIRVFKNNEKNGVAYPKNQPMRIYSSLWEADDWATEGGRVKIDWSNAPFKASYRNFNDQSSCSRTSSSKWVTCEPNSNSWMWTTLNPAQYGKMMWVQRDFMIYNYCTDFKRFPQGLPKECKL

>AtXTH26

MAGLQAKTLMFVLAAALATLGRTFVEADFSKNFIVTWGKDHMFMNGTNLRLVLDKSAGSAIKSKVAHLFGSVEMLIKLVPGNSAGTVAAYYLSSTGSTHDEIDFEFLGNATGQPYTIHTNLYAQGKGNREQQFRPWFNPTNGFHNYTIHWNPSEVVWFVDGTPIRVFRNYESEGIAYPNKQGMKVFASLWNAEDWATQGGRVKTNWTLAPFVAEGRRYKARACLWKGSVSIKQCVDPTIRSNWWTSPSFSQLTASQLTKMQKIRDGFMIYDYCKDTNRFKGVMPPECSKKQF

>AtXTH24

MSPFKIFFFTTLLVAAFSVSAADFNTDVNVAWGNGRGKILNNGQLLTLSLDKSSGSGFQSKTEYLFGKIDMQIKLVPGNSAGTVTTFYLKSEGSTWDEIDFEFLGNMSGDPYTLHTNVYTQGKGDKEQQFHLWFDPTANFHTYSILWNPQRIILTVDDTPIREFKNYESLGVLFPKNKPMRMYASLWNADDWATRGGLVKTDWSKAPFMASYRNIKIDSKPNSNWYTQEMDSTSQARLKWVQKNYMIYNYCTDHRRFPQGAPKECTTSS

>AtXTH18

MKLSCGTSFAFLIMFLFAAQSMHVYAGSFHKDVQIHWGDGRGKVRDRDGKLLSLSLDKSSGSGFQSNQEFLYGKAEVQMKLVPGNSAGTVTTFYLKSPGTTWDEIDFEFLGNLSGHPYTLHTNVYTKGSGDKEQQFHLWFDPTVNFHTYCITWNPQRIIFTVDGIPIREFKNSESIGVPFPTKQPMRLYASLWEAEHWATRGGLEKTDWSKAPFTAFYRNYNVEGCVWANGKSSCPANSSWFTQQLDSNGQTRMKGVQSKYMVYNYCNDKRRFPRGVPVECS

>AtXTH19

MKSFTFLILFLFAAQSISVYAGSFHKDVKIHWGDGRGKIHDNQGKLLSLSLDKSSGSGFQSNQEFLYGKAEVQMKLVPGNSAGTVTTFYLKSPGTTWDEIDFEFLGNISGHPYTLHTNVYTKGSGDKEQQFHLWFDPTANFHTYCITWNPQRIIFTVDGIPIREFMNAESRGVPFPTKQPMRLYASLWEAEHWATRGGLEKTDWSKAPFTAYYRNYNVEGCVWVNGKSVCPANSQWFTQKLDSNGQTRMKGVQSKYMVYNYCSDKKRFPRGVPPECS

>AtXTH07

MVVSLFSSRNVFYTLSLCLFAALYQPVMSRPAKFEDDFRIAWSDTHITQIDGGRAIQLKLDPSSGCGFASKKQYLFGRVSMKIKLIPGDSAGTVTAFYMNSDTDSVRDELDFEFLGNRSGQPYTVQTNVFAHGKGDREQRVNLWFDPSRDFHEYAISWNHLRIVFYVDNVPIRVYKNNEARKVPYPRFQPMGVYSTLWEADDWATRGGIEKINWSRAPFYAYYKDFDIEGCPVPGPADCPANSKNWWEGSAYHQLSPVEARSYRWVRVNHMVYDYCTDKSRFPVPPPECSAGI

>AtXTH05

MGRLSSTLCLTFLILATVAFGVPPKKSINVPFGRNYFPTWAFDHIKYLNGGSEVHLVLDKYTGTGFQSKGSYLFGHFSMHIKMVAGDSAGTVTAFYLSSQNSEHDEIDFEFLGNRTGQPYILQTNVFTGGAGNREQRINLWFDPSKDYHSYSVLWNMYQIVFFVDDVPIRVFKNSKDVGVKFPFNQPMKIYSSLWNADDWATRGGLEKTNWEKAPFVASYRGFHVDGCEASVNAKFCETQGKRWWDQKEFQDLDANQYKRLKWVRKRYTIYNYCTDRVRFPVPPPECRRDRDI

>AtXTH20

MVSFCGRRFAFLIIFLFAAQYERVYAGSFHKDVQIHWGDGRGKILDNVGNLLSLSLDKFSGSGFQSHQEFLYGKVEVQMKLVPGNSAGTVTTFYLKSPGTTWDEIDFEFLGNISGHPYTLHTNVYTKGTGDKEQQFHLWFDPTVDFHTYCIIWNPQRVIFTIDGIPIREFKNSEALGVPFPKHQPMRLYASLWEAEHWATRGGLEKTDWSKAPFTAFYRNYNVDACVWSNGKSSCSANSSWFTQVLDFKGKNRVKWAQRKYMVYNYCTDKKRFPQGAPPECS

>AtXTH12

MAAFATKQSPLLLASLLILIGVATGSFYDSFDITWGAGRANIFESGQLLTCTLDKTSGSGFQSKKEYLFGKIDMKIKLVPGNSAGTVTAYYLSSKGETWDEIDFEFLGNVTGQPYVIHTNVFTGGKGNREMQFYLWFDPTADFHTYTVLWNPLNIIFLVDGIPIRVFKNNEANGVAYPKSQPMKIYSSLWEADDWATQGGKVKTDWTNAPFSASYRSFNDVDCCSRTSIWNWVTCNANSNSWMWTTLNSNQLGQLKWVQKDYMIYNYCTDFKRFPQGLPTECNLN

>AtXTH13

MAAFTTKQSLLLLSLLLLISLSAGSFYDNFDITWGNGRANIVESGQLLTCTLDKISGSGFQSKKEYLFGKIDMKMKLVAGNSAGTVTAYYLSSKGETWDEIDFEFLGNVTGQPYVLHTNVFTGGKGNREMQFYLWFDPTADFHTYTVLWNPLNIIFLVDGIPIRVFKNNEANGVAYPKSQPMKIYSSLWEADDWATQGGKVKTDWTNAPFSASYKSFNDVDCCSRTSLLNWVTCNANSNSWMWTTLNSNQYGQMKWVQDDYMIYNYCTDFKRFPQGLPTECNLN

>AtXTH25

MDRSTFILSLLFTLTVSTTTLFSPVFAGTFDTEFDITWGDGRGKVLNNGELLTLSLDRASGSGFQTKKEYLFGKIDMQLKLVPGNSAGTVTAYYLKSKGDTWDEIDFEFLGNLTGDPYTMHTNVYTQGKGDREQQFHLWFDPTADFHTYSVLWNPHHIVFMVDDIPVREFKNLQHMGIQYPKLQPMRLYSSLWNADQWATRGGLVKTDWSKAPFTASYRNFRADACVSSGGRSSCPAGSPRWFSQRLDLTAEDKMRVVQRKYMIYNYCTDTKRFPQGFPKECRH

>AtXTH22

MAITYLLPLFLSLIITSSVSANFQRDVEITWGDGRGQIKNNGELLTLSLDKSSGSGFQSKNEYLFGKVSMQMKLVPGNSAGTVTTLYLKSPGTTWDEIDFEFLGNSSGEPYTLHTNVYTQGKGDKEQQFKLWFDPTANFHTYTILWNPQRIIFTVDGTPIREFKNMESLGTLFPKNKPMRMYSSLWNADDWATRGGLVKTDWSKAPFTASYRGFQQEACVWSNGKSSCPNASKQGTTTGSWLSQELDSTAQQRMRWVQRNYMIYNYCTDAKRFPQGLPKECLAA

>AtXTH06

MAKIYSPSFPGTLCLCIFTLLTLMFIRVSARPATFVEDFKAAWSESHIRQMEDGKAIQLVLDQSTGCGFASKRKYLFGRVSMKIKLIPGDSAGTVTAFYMNSDTATVRDELDFEFLGNRSGQPYSVQTNIFAHGKGDREQRVNLWFDPSMDYHTYTILWSHKHIVFYVDDVPIREYKNNEAKNIAYPTSQPMGVYSTLWEADDWATRGGLEKIDWSKAPFYAYYKDFDIEGCPVPGPTFCPSNPHNWWEGYAYQSLNAVEARRYRWVRVNHMVYDYCTDRSRFPVPPPECRA

>HvXTH22

MLRGSLRWLLVLAVVVAASAGKAGRGLHRDFDAVWGKRNARFFDEGRVVELALDRETGSRLQSKDRYLFGRFDLDIRLVAGESAGTITSFYICTGGARHDEVDFEFLGNVSGEPYILHTNIFSDGKGEREQQFVLWFDPTADFHTYSILWNPLNIILYIDGTPIRVFKNNEANGVPFPTRQPVHVFASIWNAEEWATQGGRVKTDWSEAPFVAAYRRFDASSACVWHGGASPTRCGGDHLPSSASSWMGQRLDWWSWMTLNWVRMNYMTYDYCADRKRYPHGFPAECIIPIGRI

>HvXTH20

MARMAVSVLAILLAWCALAAASFDKEFDITWGDGRGKILNNGQLLTLALDKVSGSGFQSKHEYLFGKIDMQLKLVPGNSAGTVTAYYLSSQGPTHDEIDFEFLGNVTGEPYTLHTNVFTQGQGQREQQFRLWFDPTNDFHTYSILWNPKHIIFMVDDMPIRDFKNLEGKGIAFPKNQPMRLYSSLWNADDWATQGGRVKTDWSHAPFSASYRGFKADACVVTAGGRPHCGASVGTDVAPGTGAAGEWYNQELDLTRQQRMRWVQSNYMIYNYCTDPKRFAQGVPAECSM

>HvXTH16

MASLSLLPAMALLLLAMAVASSDAQPSPGYYPSSRFRPVAFNRGYSNKWGPQHQTVSGDHSAITIWLDRTCGSGFKSKHAYRNGYFATRIKLPAGYTAGTNTAFYLSNNEAHPGFHDEVDMEFLGTIPGEPYTLQTNVYVRGSGDGRIIGREMRFHLWFDPTAGFHNYAILWNPDAITFFVDDVPIRRYERKTELTFPDRPMWAYGSIWDASDWATDHGRHRADYRYQPFVARFDRFVVAGCGPGAPPSCRPVRASPVGTGLTRQQYAAMRWAQQRHMVYYYCQDFRRDRSLTPEC

>HvXTH19

MARMAVSVLSILLATCALAAASFDKEFDVTWGDGRGKILNNGQLLTLGLDKVSGSGFQSKHEYLFGKIDMQLKLVPGNSAGTVTAYYLSSQGPTHDEIDFEFLGNVTGEPYTLHTNVFTQGQGQREQQFRLWFDPTNDFHTYSILWNPKHIIFMVDDMPIRDFKNLEGKGIAFPKNQPMRLYSSLWNADDWATQGGRVKTDWSHAPFSASYRGFKADACVVTAGGRPRCGASVGTDVAPGTGAAGEWYNQELDLTRQQRMRWVQSNYMIYNYCTDPKRFAQGVPAECSM

>HvXTH15

MASSVRQPWLLLLLVLLPVMATAAVFDDNYAPTWGADGYHLVDQGTEIRLTMDRNSGAGFHSKSTYGSGFFHMRIKVPGGYTAGVVTAFYLASETPYDGSDRDEVDFEFLGNVDGENITLQTNVFVNGDGDREQRLSLWFDPAADFHEYKILWNPYHLVILVDDVPIRVLRNLTGQVAEYEFPAKRMAVRASLWDGSDWATDGGRTKIDWGRAPFTAGFRGFDVDACDNASSTPCDSTDLWWNARRHRRLSVREQAAYENVRRTYMNYDYCADKDRFQNGKLPVECSYTT

>HvXTH11

MASSSSCPPPSPRPSRLLPVLVATVVLLGRGGEARQPAPLHGVVRSMAFDEGYTQLFGSGNLALRREGKRVHLALDESTGSGFASQDRFLHGFFSAAVKLPADYAAGVVVAFYLSNADVYEKTHDELDFEFLGNVRGREWRVQTNVYGNGSTGAGREERYDLPFDPTDDFHHYSILWTQHRIIFYVDETPIREVVRTEAMGAAFPSKPMSLYATIWDGSAWATLGGRYRANYKYAPFVAEFGDLVLHACPVNRIYHSAAAACGTPWYEPVAAALSGEQRASMSAFRRGHMSYSYCHDRRRYPVALSECDVAVLPRLFGPDGMKYGGDRRHRRGGRGRRSDVVM

>HvXTH03

MKAPSGLGLAYKKAVSCALCFAPDQSISTLLHSPPLCLPLCGFAVSRPTFVVGGTVFVSSWGWGAMGPWRRPCVGALLACAAIAASCCCFQLQGADAAASPSFGDNFEITGAKDHVKTSPDGQTWYLSLDNKTGVGFQTKQKYLFGWFSMKLKLVGNDSAGVVTAYYMCSDLDAAPERDELDFEFLGNRTGEPYIIQTNVYRSGVGGREMRHSLWFDPTADFHSYSILWNPKQIVFFVDKVAIREYRNSAKPNKFFPIMKPMYVFSSIWNADDWATRGGLEKTDWTKGPFVSSYSDFTADACAWPSGPAPPACAAATGDSWWDQPPAWALDDGQRRDSGWVARNLVIYDYCGDRKRFPTVPEECALRTTTS

>HvXTH02

MGKPGALVPVVALAFALVLGLELVSGGNFYEECDATWEPQNCWTYDGGNSLSLALVSNSSGLSSTSQLLYFSWSSPLNESMCGCSGSMIRSKRQFIYGTVSTMIQLVKGDSAGTVTTYYTSSVGDDHDEIDFEFLGNETGQPYTLHTNVYAAGVGGKEMQFRPWFDPTDGYHNYTIAWTPCAVVWYVDGAPIRAFRNYERTHGVAFPTTRPMHAYSSIWAAEDWATQGGRVRADWTRAPFVASYRGIDLDICECYGGDCVYTCAGAFRGCGGLTGDQRGKMQWVQDNYRIYDYCADHEAGKVPGVECSLPQY

>HvXTH14

MAPRSDLLAALALALLAASVLSTGAKADFDDQFEVIGDRDHIGYRDDGNDKGQEFSLELDQESGSGFKSKAKYLFGEFQVRMKLVDGNSAGTVTSFYLTSGESATHDEIDIEFMGNSSGDPYVMNTNVWASGDGKKEHQFYLWFDPSADFHTYKITWNPKNIIFEVDGVPVRTFKKYDGLPFPSARPMTVHATLWDGSYWATQHGTVKIHWRHDPFVVPYQGYHANGCVHDKATNKTSCPAGSDAWMHRELDDGELSTVAWAERNCLSYNYCADGWRFPKGFPGECGRK

>HvXTH12

MEMTARFLAAAAACVWLAAAASAFDVPTVAFEEGFSPLFGDGNLVRARDDRAARLLLDRRSGSGFISSDYYLHGFFSASIKLPRDYTAGVVVAFYLSNGDVYEKTHDELDFEFLGSRWGGQWRVQTNVYGNGSTSRGREERYLLPFDPTLAAHRYSILWAPTHIIFYVDDTAIREVVRHPGMGGDFPAKPMAAYATIWDGSAWATEGGKYKVNYKYAPFASDFSDLSLRGCRVADPASPALRLAGGDGCDLLGLMTADYAVMTPQKRAAMRAFRARRMTYTVCYDAARYAAGPFPECDNSDEERGTFWAWGESKTVVMKTRGRGRRGRGSRAGAGARGRAGAASS

>HvXTH10

MAMMQIRRPHDAISHLMVIVVGAVILLQGEAQPSPGYYPSSKVSSTPFSQWYSTLWGPQHQSLSPDQTALTLWMDRSSGSGFKSKRSYRNGYFGVSMKVQPGYTAGVNTAFYLSNNEVYPGYHDEIDVELLGTVPGEPYTLQTNVYVRGTGDAHPIVGREMRFHLWFDPAAAFHHYAVLWNPDEIVFLVDDVPVRRYQKKVEATFPEREMWAYGSVWDASDWATDGGRYRSDYRYQPFVSGFKDFKVAGCEVGAPASCRPVPAGPGGGLSAQQSAAMSWAQQRAMVYYYCQDGSKDRSNYPEC

>HvXTH13

MAPSLPSSSSCWHSALLVAMLVLVVVMDQVAMAYLDDDIEVVWGDDHSFFYMDDAGDDEILALCLDETHGSGFHTKEAYLYARFDVDLMLVPDNSAGTVTTLYLMPEDVPWDYHDEVDLEFLGNVTGEPYTLHTNIFANGVGNREEQFRLWFDPTADFHTYSIDWNPKRITILVDGVPIRSFRNNEEHGVAFPTWQKMRLHGSLWNADDWATQGGRVKTDWSGAPFFARYRNLRASWCRPSPGVAWCGDEPPGSTWFERGLDAAALRRARDAHMIYDYCKDLQRYKGSGLPKECVVD

>HvXTH17

MARMGASVLVILLASCALAAASFDKEFDVTWGDGRGKILNNGQLLMLGLDKVSGSGFQSKREYLFGKIDMQLKLVPGNSAGTVTAYYLSSQGPTHDEIDFEFLGNVTGEPYTLHTNVFTQGQGQREQQFRLWFDPTNDFHTYSILWNPKHIIFLVDDMPIRDFRNMEGKGIAFPKNQPMRLYSSLWNADDWATQGGRVKTDWSHAPFSASYRGFKADACVVTVGGRPRCGASIGTDAAPGTGGAAAVGDWYNQELDLTRQQRMRWVQSNYMIYNYCTDPKRVAKGVPAECSM

>HvXTH21

MASGPSRTVPCSVLPLLLLLAGVARAAGNFYQDVDITWGDGRGKILGGGDLLTLSLDRASGSGFQSKNQYLYGRFDMQIKLVPGDSAGTVATFYLSSQGSAHDEIDFEFLGNASGQPYTVHTNVYSQGKGGREQQFRMWFDPTADFHTYSVLWNPTHILFYVDGTPIREHRNREAATGVPYLRSQAMRVYASVWDAEEWATQGGRVRTDWSRAPFVASYKGLAASGCASQDAAACANSNGAWMYQELDATALDRLQWVQKNYMIYNYCTDTWRFKDGAPPECASK

>HvXTH24

MGQARAYLLASLAAFYLVALAIPQVTADMTDEVNLLWGNCKVQRDGTGRQTVAMSLDRWTTSGFSSKIKYLFGRIDMEIKLMPGNSAGTVTTFYMMSEGPWQFHDEIDLEFLGNSTGNPYTLHTNVYARGVGSREKGYRLWFDPSQDFHTYSIIWTQQYIRFLVDNKLIRQIKNKMMNGSPYPNYQPMRVFSTIWNADDWATQGGRVKTDWTQAPFTAYFRNYKATSCSQGQNSNVCGQSSPNGLFNQQQDQMQQQQVKEVDAKYKVYDFCDDSKRRIGSSEDCQSQ

>HvXTH23

MRTVELGIVAMACLVAVARAGNFFQDSEMSWGDGRGKVVDGGRGLDLTLDKTSGSGFQSKSEYLFGKIDMQIKLVPGNSAGTVTTFYLSSQGTAHDEIDFEFLGNVTGEPYTLHTNVFAQGQGQREQQFRLWFDPTKAFHTYSIIWNPQHVIFAVDGTAIRDFKNHEARGVSFPKSQPMRLYASLWNADDWATQGGRVKTDWSKAPFVASFRNFNADACVMSGGAQRCPAGTMEASAAGGGSWWNQELSGMGYRRMRWVQRKFMIYNYCTDPKRVAQGVPAECKLR

>HvXTH18

MARMGASVLSILLASCALAAASFDKEFDVTWGDGRGKILNNGQLLTLGLDKVSGSGFQSKHEYLFGKIDMQLKLVPGNSAGTVTAYYLSSQGPTHDEIDFEFLGNVTGEPYTLHTNVFTQGQGQREQQFRLWFDPTNDFHTYSILWNPKHIIFMVDDMPIRDFKNLEGKGIAFPKNQPMRLYSSLWNADDWATQGGRVKTDWSHAPFSASYRGFKADACVVTAGGRPRCGASMGTEAAPGTGASGAAGEWYNQELDLTLQQRMRWVQSNYMIYNYCTDPKRVAKGVPAECSM

>HvXTH01

MARPSFSLHLCLAVLALAAAASEAGFYDQFDVVGSGNNVRVNDDGIAQQVALTLDQGNGGSGFSSKDKYLYGEFSVQMKLIGGNSAGTVTSFYLTSGEGDGHDEIDIEFMGNLSGDPYVMNTNVWASGDGKKEHQFYLWFDPTADFHTYKIVWNPKNIIFQVDDVPVRTFKKYDDLPYPSSQPMTVHATLWDGSYWATRHGDVKIDWTQAPFVVNYRGYTSNGCVSNGGSSACPAGSDAWMSTELDAKALGTVAWAESKYMSYDYCTDGWRFPNGFPAECSRRN

>HvXTH06

MAQRFLAVLAVALALSQVASAKSWLDKRFNTDGTVRTGYDASGQQVVMLNLNQQSGAAGFNSKQQYLYGEFSIQMKLIPGNSAGTVSCFYLSSGDDEWRDEIDMEFMGNSSGHPVVLNTNVWANGDGKKEHQFDLWFDPAADYHTYTIIWNPENILFKVDNLFIRSFKRFAGLPYPTSKPMRLHATLWDGSYWATEKGKIPINWSNAPFVVSYRNYYANACVSGGACHAGSDRWMRKQLDGDEWGTVKWAERSYMRYNYCEDGYRFPQGLPAECNRY

>HvXTH05

MARRLLAVLAVALALLQAASAKSWLDKRFNTDGTVRTGYDASGQQVVMLNLNQQSGAAGFNSKQQYLYGEFSIQMKLIPGNSAGTVSCFYLSSGDDEWRDEIDMEFMGNSSGHPVVLNTNVWANGDGKKEHQFDLWFDPAADYHTYTIIWNPENILFKVDNLFIRSFKRFAGLPYPTSKPMRLHATLWDGSYWATEKGKIPINWSNAPFVVSYRNYYANACVSGGACHAGSDRWMKKQLDGAEWGTVKWAERSYMRYNYCEDGYRFPQGLPAECNRY

>HvXTH07

MSNTSTLSSGDGDGHDEIDMEFMGNSSGPGHPVVLNTNVWVNGDGKKEHQFDLWFDPAADYHTYTIIWNPENILFKVDNLFIRSFKRFAGIPYAGSKPMRLHATLWDGSYWATEKGKVPIDWSNAPFNVLYKNYYANACASGGACHAGSDGWMNRQLDGSEWGTVKWAEQNYMRYNYCADGYRFPQGFPAECSRY

>HvXTH04

MAPALPCSRPKLLLLCVALAFLLAVDVGRADIYKDIQIIWSADHTYYFMDGDSEALALSLDFNRGSAFKSNDMYLYARIDIDIKLVEGNSAGTVCTVYTISEGPWDIHDEIDLEFLGNSTGEPYTLHTNIFAYGVGGREQQFKLWFDPSAEYHTYSIVWNPRRITIEVDGVTIRSYDNNEEHGVPFPAWQQQRVYGSLWNADDWATQGGRVKTDWKLAPFVSYYRNYNITYCRPSPGVSWCGAEPAGSPVFNLAPKARADMQWVRDMGYVIYDYCTDRSNRYNDTTRPKECSLPPRP

>HvXTH09

MACHFLLAVLLASSSWVAASSGAAADDVMVPRPTTAAALTFREGYTQLFGDSNLRLHGDGKRVHISLDERTGSGFASQGAYFHGFFSASIKLPSDYAAGVVVAFYVSNGDVYEKTHDELDFEFLGNVRGKEWRVQTNVYGDGSTAVGREERYGLWFDPTHDFHRYAILWTNRTIVFYVDGTPIREVVRSEAMGAQFPSKPMSLYATIWDGSSWATSGGRYKVEYKYAPYVAEFTDLELRGCASHDRAQPASCEPEGMPARQRAAMERVRARHMTYGYCYDRARYPAPLPECRVGAEAAMYLPSGEARSSDRRRHGKRHRRADSAL

>HvXTH08

MKATAGALLAVVATVLLRGIAAAPPRKPVDVPFEKNYVPTWAEDHIHYVNGGREVQLSLDKTTGTGFQTRGSYLFGHFSMHIKLVGGDSAGTVTAFYVPSQNSEHDEIDFEFLGNRTGQPYILQTNVFSGGKGDREQRIYLWFDPTKDYHSYSVLWNLYMIAFFVDDTPIRVFKNSKDLGVRYPFDQPMKLYSSLWNADDWATRGGREKTDWSKAPFVASYRGFHVDGCEASAEAKLCATQGARWWDQPEFQDLDAAQYRRLAWVRKEHTIYNYCTDRERYAAMSPECKRDRDV

>LcXTH06

MGAPPKKPVDVPFQKNYAPTWASDHIKYINGGSEVRLVLDKYSGTGFQSKDSYLFGHFSMKIKMVGGDSAGIVTAFYLSSEGTEHDEIDFEFLGNRTNQPYILQTNVFTGGKGDREQRIYLWFDPTKHYHSYSILWNMHQIVFFVDEVPIRIFKNSKDLGVKFPFNQPMKIYSSLWNADDWATRGGQEKTDWSKAPFVASYTTFHVDGCDASQSAMVCTTRGRMWWDQKAFQDLDGPQYRKLMWVRKKYTIYNYCDDRSRYPSVPPECVRDRDI

>LcXTH09

MSKLSPPTLSSLHAFLLFTFFLTVSADFTSDVDLTWGGQRGTISGGGRLLQLSLDKASGAGFQSKHEYLYGKIDMQIKVVPGNSAGTVTAYYLSSQGPTHDEIDYEFLGNETGQPYILHTNVFTQGKGNREQQFYLWFDPRADFHTYSILWNQQQIIFSVDGTPIRVFRNAQGVGIPYPKDQPMRLYSSLWNADDWATRGGLVKTDWAHAPFIASYRSFSADACVWSAGRSLCSPTKARWWAQSLDSNGAQKLKWVQKNYMIYDYCRDTKRFPQGPPKECSLSGL

>LcXTH10

MLLPILLIASSLTAAYGGNFHQDFDVTWGDGRAKIMNYGQLLTLSLDKASGSGFQSKNEYLFGKIDMQLKLVPGNSAGTVTAYYLKSQGSNWDEIDFEFLGNLSGDPYILHTNIFTQGKGNREQQFYLWFDPTRNFHTYSILWNPQHVIFSVDGRPIRVFKNGEAIGVPFLNKQPMRIYSSLWNADDWATRGGLIKTDWSKAPFTAAYRNFNANACVWSSGRSSCDSGSSSSLSNAWLTQELDVTNQRRLRWVRKNFMIYNYCTDLKRFPQGLPAECKFP

>LcXTH11

MVSPSPCVSSSIGNIMLVIFLLTCSLMVASGTNFYRDVDITWGDSRAKILNNGKVLALTLDKDSGSGFRSKNEFLFGKIDMQLKLVPGNSAGTVTAYYLSSQGKTWDEIDFEFLGNLSGDPYTVHTNVFTQGKGNREQQFQLWFDPTKAFHTYSILWNPQHIILYVDDIPIRIFKNFESKGVPFLNKQPMRIYSSLWNADNWATRGGLIKIDWSNAPFTAAYRNFKARACIMLSGISSCASQSPSLPSMAWMTQELDSASQEKLRWVQKNYMIYNYCADPNRFPQGLPPECGFA

>LcXTH03

MAPLFPLVLFLMFSSTNAENPPSPGYYPSSKFRSLGFYQGFRNLWGSQHQSVSQDQSLTIWLDRTSGSGFKSTRPFRSGYFGASIKLQPGYTAGVITAFYLSNNEVHPGNHDEVDIEFLGTTPGKPYTLQTNVYIRGSGDGRIIGREMKFHLWFDPTTDFHKYAILWGPNEIIFFVDDVPIRRYPRKSVSSFPMRPMWVYGSIWDASSWATENGKYKADYNYQPFVAHYTRFITSGCSAYSPARCYPVSVSPSGSGGLSSQQTMAMQWTQRNYMVYNYCQDPSRDHGLTPECWA

>LcXTH13

MASSSSTIHIMLPLLLLASYLIPAYAANFYQDFDSTFGDGRVRILDNGELLTLSLDKASGSGFKSKNEYLFGKIDMQLKLVPGNSAGTVTAYYLTSQGPTWDEIDFEFLGNLSGDPYILHTNVFTQGKETESNSFTSGSTQLPTSIHTPSSGIPNTSYQWQVYFSYNCLCGPPFRFSVDGTPIREFKNMESKGVQYPKNQPMRIYSSLWNADDWATRGGLVKTDWSQAPFTASYRNFNANACVWSSGTSSCDSKTQSTDNGWLNEELDSTGEERLRWVQTNYMIYDYCSDSKRFPQGFPPECNTTQQN

>LcXTH14

MASSSSNVLIMLPLLLLASSLIPAYAANFYQDFDSTFGDGRVRILNNGELLTLSLDKASGSGFKSKNEYLFGKIDMQLKLVPGNSAGTVTAYYLTSQGPTWDEIDFEFLGNLSGDPYILHTNVFTQGKGNREQQFYLWFDPTADFHTYSILWNPQHIIFSVDGTPIRKFKNMEANGVQYPKNQPMKIYSSLWNADDWATRGGLVKTDWSQAPFTASYRNFNANACVWSSGASSCDSKAQPTNNGWLNEELDSTGEERLRWVQKNYMIYDYCSDSKRFPQGFPTECNTTQQN

>LcXTH15

MPKLPLTTLFSLHGFLLTLFVVVSADFTSQIDLSWGGDRAKISEDGRLLQLSLDAVSGAGFQSKQEFLYGKIDMQIKVVPGNSAGTVTAYYLASKGTTQDEIDFEFLGNVSGQPYILHTNVYTQGKGEREQQFYLWFYVDDIPIRVYKNNEAKGVPYPKSQPMGVYSTLWDADNWATRGGLEKTDWSKAPFYAYYKDFDIEGCTVPGPADCTLKSSNWWDGPLYQQLNPIQARKYRWVRVNHMIYDYCTDKPRNPVPPPECLAGI

>LcXTH16

MLIQPSIAKPATFTQDFRVTWAQSHIKQMENGAAIQLLLDQSSGCGFASKTKYVFGRVSMKIKLIPGDSAGTVTAFYLNSDTKEIHDELDFEFLGNRSGQPYTVQTNVYAHGKGDREQRVNLWFYVDDVPIRVYKNNEARGVPYPKSQPMGIYSTLWDADNWATRGGLEKTDWSKAPFYAYYKDFDIEGCTVPGPAGCTTKSNNWWDGPLYQQLNPIQARKYKWVRVNHMVYDYCTDKHRNPVPPPECLAGI

>LcXTH17

MVASATNFHRDFDITWGGDRAKILNNGKRLTLSLDKTSGSGFRSKHSYLFGDISMQLKLVPGNSAGTVTAFYLSSSGPKWDEIDFEFLGNLSGDPYTIHTNVFTQGKVSLWMAFQSESSKMKNPWASQFPNKQPMRIYSSLWNADNWATRGGRIKTDWTKSPFIASYRNFKARACVWSSHGASSCESKSPSSVSDVWLKQELDSRSKQKLRWVRKHYMIYDYCTDSKRFAQGFPPECRYAALV

>LcXTH18

MLLFLLYASSLLATHASNFYQDFEVTWGGDGRTKILNNGELLTLSLDKASGSGFRSKNEYLFGKFDMQLKLVPGNSAGTVTAYYLSSEGPTHDEIDFEFLGNLSGEPYIVHTNVYTEGKGEREQQFYLWFDPTKDFHTYSFLWNPLHVLFYVDGTPMREFKNAEPSIRVPYPKNQPMRIYSSLWNADDWATRGGLIKTDWTQAPFTASYRNFQADACVWSSGVSSCASKLWPSSSNYTWMTLDLDSMARQRLRWVQKNYMVYNYCSDVKRFPQGLPPECSINSTA

>LcXTH19

MLPFLLLASSIIPAYAANFYQDFDTTFGDGRVRILNNGELLTLSLDKASGSGFKSKNEYLFGKIDMQLKLVPGNSAGTVTAYYLSSQGPTWDEIDFEFLGNLSGDPYILHTNVFTQGKGNREQQFYLWFDPTADFHTYSILWNPQHIIFSVDGTPIRKFKNLESNGVQYPKNQPMRIYSSLWNADDWATRGGLVKTDWSQAPFSASYRNFNANACVWSSGASSCDSKAHSTDNGWLTQELDSTGEERLRWVQKNYMIYNYCGDSKRFPQGFPPECNNTQQN

>LcXTH20

MPPMASSSSNVLIMLPLLLLASSMIPAYAANFYEDFDTTFGDGRVRILNNGELLTLSLDKASGSGFKSKNEYLFGKIDMQLKLVPGNSAGTVTAYYLTSQGTTWDEIDFEFLGNLSGDPYILHTNVFTQGKGNREQQFYLWFDPTADFHTYSILWNPQHIIFSVDGTPIRKFKNMESKGIAYPKNQPMRIYSSLWNADDWATRGGLVKTDWSQAPFTASYRNFNANACVWSSGTSSCDSKAQSTNNGWLTQELDSTGEERLRWVQKNYMIYNYCNDSKRFTQGFPPECNNTQQN

>LcXTH21

MASSPSTIHIMLPLLLLASYLIPAYAANFYQDFDSTFGDGRVRILNNGELLTLSLDKASGSGFKSKNEYLFGKIDMQLKLVPGNSAGTVTAYYLTSQGPTWDEIDFEFLGNLSGDPYILHTNVFTQGKGNREQQFYLWFDPTADFHTYSILWNPQHIIFSVDGTPIREFKNMESNGVQYPKNQPMRIYSSLWNADDWATRGGLVKTDWSQAPFTASYRNFNANACVWSSGTSSCDSKTQSTNNGWLNEELDSTGEERLTWVQKNYMIYDYCSDSKRFPQGFPPECNNTQQN

>LcXTH22

MASSSFKIFIILPLLLLASYLKSAYAANFYQDFDSTFGDGRVRILNNGELLTLSLDKASGSGFKSKNEYLLGKIDMQLKLVPGNSAGTVTAYYLTSQGPTWDEIDFEFLGNLSGDPYILHTNVFTQGKGNREQQFYLWFDPTADFHTYSILWNPQHIIFSVDGTPIRKFKNLESKGVQYPKNQPMRIYSSLWNADDWATRGGLVKIDWSQAPFTASYRNFNANACVWSSGASSCDSKAQSPNNGWLNEELDSTGEERLRWVQQNYMIYNYCSDLKRFPQGFPPECNITQQN

>LcXTH23

MLSFLLLAFSLIPAYAANFYQDFDTTFGDGRFRILNNGDLLTLSLDKWSGSGFKSKNEYLFGKIDMQLKLVPGNSAGTVTAYYVSFNATVQFWFAIFIEVIALQLTSQGPTWDEIDYEFLGNLSGDPYILHTNVFTQGKGNREQQFYLWFDPTADFHTYSILWNPQHIIFSVDGTPIRKFKNMESNGVQYPKNQPMRIYSSLWNADDWATRGGLVKTDWSQAPFTASYRNFNANACVWSSGVSSCDSKTQSTNNGWLNEELDSTGEERLKWVQKNYMIYDYCSDSKRFPQGFPTECNITQQN

>LcXTH24

MASSSSTIHIMLPLLLLASYLIPAYAANFYQDFDSTFGDGRVRILNNGELLTLSLDKASGSGFKSKNEYLFGKIDMQLKLVPGNSAGTVTAYYLTSQGPTWDEIDFEFLGNLSGDPYILHTNVFTQGKGNREQQFYLWFDPTADFHTYSILWNPQRISVSHHGTPIRKFKNMESKGVQYPKNQPMRIYSSLWNADDWATRGGLVKTDWSQAPFTASYRNFNANACVCSSGTSSCDSKTQSTNNGWLNEELDSTGEERLKWVQKNYMIYDYCSDSKRFPQGFPTECNITQQN

>LcXTH25

MSAYAGNFYQEFDVTWGDGRGKILNNGELLTLSLDKYSGSGFQSKNEYLFGKIDMQLKLVPGNSAGTVTAYYMRSQETAWDEIDFEFLGNLSGDPYIVHTNVFTQGKGNREQQFYLWFDPTADFHTYSILWNPRHILFSVDNTPIREFKNSESIGVPFPKNQSLRIHSSLWNADDWATRGGLVKTDWSQAPFTASYRNFSANACVWSSGQSSCSSNSTSTADNGWLTEELDSTGHERIKWVQQNYMIYNYCTDTKRFPQGLPAECSSATSP

>LcXTH02

MASFLTVLCLSSFFPYVIMGAPPRTPIDVPFQRNYMPTWAYDHIKYFNGGTEVQLTLDKYTGTGFQSKGSYLFGHFSMRIKMVPGDSAGTVTAFYLSSQNSEHDEIDFEFLGNRTNQPYILQTNVFTGGKGDREQRIYLWFDPTKDYHSYSILWNMYQIVFFVDDVPIRVFKNCKDLGVRFPFNQPMKLYSSLWNADDWATRGGLEKTDWSKAPFVASYRGFHIDGCEESVRATFCSTQGKRWWDQKEFQDLDGLQYRKLIWVRQKYTIYNYCTDRSRAPTMPPECQRDRDV

>LcXTH12

MSIHTSMGHNTLSLLLLLSFSLAVAARPATFLQDFRITWSDTHIKQMEGGDAIQLTLDQSSGCGFASKNQYLFGRVSMKIKLIPGDSAGTVTAFYMNSDTDTVRDELDFEFLGNRTGQPYTVQTNVYAHGKGDREQRVNLWFDPAADFHTYSILWNHYQVVFSVDDVPIRVYRNNEARGLPFPKVQPMGIYSTLWEADDWATRGGLEKIDWSKAPFYAYYKDFDIEGCLAHGPASCASNPSNWWEGPAYQQLDALQARRYRWVRMNHMIYDYCTDKSRYPGHATGMRRQRLILFSLDFLCIPLVYMRLCTKISRLPLPVSENHIHPH

>LcXTH04

MARWAAMHSVNANVLLLCLATLLNIAFSANFSELFQPSWAPDHIMPQGDTLQLKLDNTSGCGFESKGKYLFGRVSVQIKLVEGDSAGTVTAFYMSSDGPYHDELDFEFLGNVSGEPYLVQTNVYVNGTGNREQRHTLWFDPTVDFHTYSLLWNRHHIRFLVDGIPIRVFTNKEDIGVPYPKNQGMGIYGSVWNADDWATQGGRVKTDWTHSPFISAFRSFEVDACEYLPETDDMVGKCREGGDYWWERPAMIGLSLHQSHQLKWVCRKHMVYDYCKDTMRFSDPPRECLG

>LcXTH01

MAASAAFSFLKLAVFFSLVFVAALGSSSSFEELFAPSWALDHVMYEGELLKLKLDNFSGAGFGSKSKYMFGRTTVQIKLVEGDSAGTVTAFYMSSDGPNHNEFDFEFLGNTTGEPYIVQTNIYVNGVGNREQRLNLWFDPTTDFHSYSILWNQRQVVFLVDETPIRVYTNKEKQGVPFPKEQPMGVYSSIWNADDWATQGGRVKTDWSHAPFIASYKGFEIDSCEALTSAPPGENTRRCSSIEGRKFWWDEPMMSELSVHQSHQLMWVHAKHLIYDYCTDSARFPAVPPECERR

>LcXTH05

MASSFYIVLCLSTLLFPFLVMAAPPRKPVDVPFSRNYVPTWAFDHINYLNGGSEVHLKLDNYTGTGFQSKGSYLFGHFSMRIKMVPGDSAGTVTAFYLSSQNSEHDEIDFEFLGNRTNQPYIVQTNVFTGGKGNREQRIYLWFDPTKAYHSYSVLWNMYQIAFFVDDVPIRVFKNSKDLGVRFPFNQPMKIYSSLWNADDWATRGGLEKTDWSKAPFVAAYQSFHIDGCEASVMSTYCATQGKRWWDQREFQDLDALQYKRLKWVRQKYTIYNYCTDRSRFPTMSPECKRDRDV

>LcXTH07

MSLSHGNSVFFGLLCASLAGIGLGSIVSVGDFNKDFFVTWSPSHVNTSADGLERSLKLDPESGSGFASNDMFLFGQIDMQIKLVPGHSAGTVVAFYLSSDQPKRDELDFEFLGNVSGQPYMLQTNVFADGSDDREERMYLWFDPTEDFHTYSVLWNIHQIVFMVDWVPIRVYKNHADKGVAFPRWQPMSLKISLWNGDSWATRGGKDKINWTKGPFIASFRKYKIDACVWSGNARFCRGESTTNWWNKPRYSTLTSAQRRLFRWARKYHLLYDYCLDNQRFQNNLPKECSLPKF

>LcXTH08

MGTSTSLTHTILPLLILTFSLFVDARPATFLQDFRITWSDSHIKQIEGGRAIQLVLDQSSGCGFASKSQYLFGRVSMKIKLIPGDSAGTVTAFYMNSDTDTVRDELDFEFLGNRTGQPYTVQTNVYAHGKGDREQRVNLWFDPAADFHTYSILWNHHHVVFSIDGVPIRVYKNNEARGIPFPKFQPMGIYSTLWEADDWATRGGLEKIDWSKAPFYAYYKDFDIEGCPKPGPASCASNPRNWWEGTAYQQLNAIEARRYRWVRMNHMVYDYCTDKSRYPVTPPECMAGI

>LcXTH26

MPISANKLLFAQCLISCVAVSISAQHGHFSPPILPRLTDLFPHLTFNGSYSEFFGGPTNIRPSEDGSRIDLILDKSSGSGFVSKDHYYYGFFSAAIKLPPDYTAGVVVAFYMTNADISPHNHDELDFELLGHEKRKEWALQTNVYGNGSSTGREEKFYLWFDPTQEYHRYSILWNEHHIVFLVDNIPIREVNHSQAMSGAYPSKPMSVYSTIWDGSKWATHGGKRPVDYKYAPFQASFTELEMEGCKWNRTMPVPLCSKSGQDDQSSSDPITGDEFVSLSQQQKVGMEQVRDRFMFYSYCADRARFSVLPPECNERKVGHLQDDDDDDGRMDDKDLP

>LcXTH27

MVGALYFFMIFSFTIFLQASATLQNLPILSFDEGYTQLFGDSNLMLLKDGKTVHISLDERTGAGFVSQDLYLHGFFSASIKLPADYTAGVVVAFYMSNGDIFEKTHDELDFEFLGNIRGKEWRIQTNVYGNGSTGLGREERYGLWFDPSEDFHKYSILWTNDQIIFYVDDIPIREIRKTEAMGGDFPSKPMSLYATIWDGSKWATSGGRYKVNYKYAPYIAEFSDLVLHGCAVDPIEQSPGCDNSHNDLNRLSDSTTLISPYQRSKMEEFRKKHMTYMYCYDRVRYPTPPPECVINPREASQFQGSDGLKFGRHRRHGKRHHRTPAAATGREATTTL

>LcXTH21(ORF)

ATGGCCTCTTCTCCTTCTACTATTCATATAATGTTGCCGTTATTACTATTAGCTTCCTATCTAATACCTGCTTATGCTGCTAATTTCTACCAAGACTTCGATTCCACATTCGGTGACGGCCGTGTCAGAATCCTAAACAATGGCGAATTACTCACTCTCTCCCTCGACAAGGCGTCTGGCTCCGGATTCAAATCCAAGAACGAATATCTCTTCGGAAAGATCGATATGCAGCTGAAGCTCGTCCCTGGCAACTCTGCTGGCACTGTTACAGCATACTATCTAACATCACAAGGACCAACATGGGATGAGATCGACTTCGAATTCCTAGGCAACCTTAGTGGAGACCCTTACATTCTCCACACCAATGTCTTCACACAAGGGAAAGGAAACAGAGAGCAACAGTTCTACCTCTGGTTCGACCCAACTGCCGACTTCCATACATACTCCATCCTCTGGAATCCCCAACACATCATATTCTCCGTAGACGGCACTCCCATCAGGGAATTCAAGAACATGGAGTCGAACGGTGTCCAGTACCCGAAGAACCAACCAATGAGGATATACTCGAGCCTCTGGAATGCCGATGACTGGGCCACCCGCGGCGGGCTCGTCAAGACCGATTGGTCGCAGGCGCCCTTCACTGCGTCCTACAGAAATTTCAACGCCAATGCATGTGTGTGGTCATCGGGCACGTCTTCATGCGATTCAAAGACTCAGTCCACCAATAACGGATGGCTGAATGAAGAGCTGGATTCGACGGGTGAAGAGAGGTTGACATGGGTGCAGAAGAATTACATGATCTATGACTATTGCAGCGATTCAAAGCGGTTTCCTCAGGGCTTCCCTCCCGAATGCAACAACACCCAACAGAATTAG
